# Comprehensive Review of Genetic Association Studies and Meta-Analyses on miRNA Polymorphisms and Cancer Risk

**DOI:** 10.1371/journal.pone.0050966

**Published:** 2012-11-30

**Authors:** Kshitij Srivastava, Anvesha Srivastava

**Affiliations:** 1 Medical Genetics Branch, National Human Genome Research Institute (NHGRI), National Institutes of Health, Bethesda, Maryland, United States of America; 2 Cancer Research Laboratory, Department of Biology, University of the District of Columbia, Washington, D. C., United States of America; Cardiovascular Research Institute Maastricht, Maastricht University, The Netherlands

## Abstract

**Background:**

MicroRNAs (miRNAs) are small RNA molecules that regulate the expression of corresponding messenger RNAs (mRNAs). Variations in the level of expression of distinct miRNAs have been observed in the genesis, progression and prognosis of multiple human malignancies. The present study was aimed to investigate the association between four highly studied miRNA polymorphisms (mir-146a rs2910164, mir-196a2 rs11614913, mir-149 rs2292832 and mir-499 rs3746444) and cancer risk by using a two-sided meta-analytic approach.

**Methods:**

An updated meta-analysis based on 53 independent case-control studies consisting of 27573 cancer cases and 34791 controls was performed. Odds ratio (OR) and 95% confidence interval (95% CI) were used to investigate the strength of the association.

**Results:**

Overall, the pooled analysis showed that mir-196a2 rs11614913 was associated with a decreased cancer risk (OR = 0.846, *P* = 0.004, TT vs. CC) while other miRNA SNPs showed no association with overall cancer risk. Subgroup analyses based on type of cancer and ethnicity were also performed, and results indicated that there was a strong association between miR-146a rs2910164 and overall cancer risk in Caucasian population under recessive model (OR = 1.274, 95%CI = 1.096–1.481, *P* = 0.002). Stratified analysis by cancer type also associated mir-196a2 rs11614913 with lung and colorectal cancer at allelic and genotypic level.

**Conclusions:**

The present meta-analysis suggests an important role of mir-196a2 rs11614913 polymorphism with overall cancer risk especially in Asian population. Further studies with large sample size are needed to evaluate and confirm this association.

## Introduction

MicroRNAs (miRNAs) are a class of endogenous, small nonprotein-coding single-stranded RNA molecules of ∼22 nucleotides in length that regulate a broad range of biologic and pathologic processes [1], [2]. Mature miRNAs regulate the expression of approximately 30% of all human genes involved in fundamental biological processes at post-transcriptional level by sequence-specific binding to 3′ untranslated regions (UTRs) of multiple target messenger RNAs (mRNAs), leading to their degradation or translational suppression [3]. To date, more than 1200 miRNA sequences have been identified in humans, although specific functions have not yet been delineated for most of them.

Cancer is eventually an outcome of chaotic expression of genes involved in developmental, cell growth and differentiation processes. Recent studies have implicated miRNAs in the genesis, progression (proliferation, migration and invasion) and prognosis of multiple human malignancies [4], including their key role in promoting cancer stem cell tumorigenicity [5]. Variations in the level of expression of distinct miRNAs (“Oncomirs”) have been observed in the development and progression of multiple human cancers and >50% of these miRNA genes are found to be located in cancer-related chromosomal regions functioning either as oncogenes or tumor suppressor genes [6]–[9]. Thus, variations in miRNA expression may promote carcinogenesis by modulating the expression patterns of essential genes involved in tumor growth and progression [10].

Single nucleotide polymorphisms (SNPs) are the most common form of variation present in the human genome. SNPs present in the miRNA gene regions can alter their expression and/or maturation leading to aberrant miRNA regulation. Many epidemiological studies have examined the association of SNPs in microRNAs with cancer susceptibility (Table 1). However, due to power considerations in single SNP studies with relatively small sample sizes, the outcomes of these studies remain contradictory rather than convincing. The present article applied a meta-analytic approach for relevant miRNA SNPs to better clarify potential associations between these SNPs and cancer. We also systematically reviewed published meta-analyses of observational studies investigating the association between miRNA polymorphisms and cancer risk to investigate their strengths and limitations.

**Table 1:** Characteristics of eligible studies in meta-analysis.

S.no.	Reference	PublicationYear	Country origin	Ethnicity	Cancer Type	N (cases)	N (controls)	Control source	Genotyping method	HWE	Matching criteria
1	Xu et al., [35]	2008	China	Asian	HCC	479	504	HB	PCR-RFLP	Yes	age/sex
2	Hu et al., [48]	2009	China	Asian	BC	1009	1093	PB	PCR-RFLP	Yes	age/area
3	Jazdzewski et al., [24]	2008	USA	Caucasian	PTC	608	901	PB	Sequencing	Yes	NR
4	Tian et al., [49]	2009	China	Asian	LC	1058	1035	PB	PCR-RFLP	Yes	age/sex/area
5	Ye et al., [23] *	2008	USA	Caucasian	EC	346	346	HB	SNPlex	Yes	age/sex
6	Catucci et al., [50]	2010	Italy	Caucasian	BC	1894	2760	PB	TaqMan and sequencing	Yes	age
7	Hoffman et al., [22] $	2009	USA	Caucasian	BC	441	479	HB/PB	SequenomMassARRAY	Yes	age
8	Peng et al., [51]	2010	China	Asian	GC	213	213	HB	PCR-RFLP	Yes	age/sex
9	Zhou et al., [52]	2010	China	Asian	CC	703	713	PB	PCR-RFLP	Yes	age/area
10	Yang et al., [53]	2010	Germany	Caucasian	BC	1217	1422	PB	TaqMan and sequencing	Yes	age
11	Xu et al., [54]	2010	China	Asian	PC	251	280	HB	PCR-RFLP	Yes	age
12	Qi et al., [55]	2010	China	Asian	HCC	361	391	HB	PCR-LDR	Yes	NR
13	Dou et al., [56]	2010	China	Asian	Glioma	670	680	HB	PCR-RFLP	Yes	age/sex/area
14	Xu et al., [57]	2011	China	Asian	HCC	501	548	PB	PCR-RFLP	Yes	age/sex/area
15	Kim et al., [58]	2010	Korea	Asian	LC	654	640	HB	PCR-FRET	Yes	age/sex
16	Christensen et al., [59]	2010	USA	Caucasian	HNSCC	484	555	PB	TaqMan	Yes	age/sex/area
17	Srivastava et al., [60]	2010	India	Caucasian	GBC	230	230	PB	PCR-RFLP	Yes	age/sex
18	Liu et al., [61]	2010	USA	Caucasian	HNSCC	1109	1130	HB	PCR-RFLP	Yes	age/sex
19	Zeng et al., [62]	2010	China	Asian	GC	304	304	HB	PCR-RFLP	Yes	age/sex
20	Sun et al., [63]	2010	China	Asian	GC	304	304	HB	PCR-RFLP	Yes	age/sex
21	Guo et al., [64]	2010	China	Asian	ESCC	444	468	HB	SNaPshot	Yes	age/sex/area
22	Yang et al., [65]	2011	Germany	Caucasian	BC	2854	3188	PB	MALDI-TOF mass spectrometry	Yes	age
23	Li et al., [66] #	2010	China	Asian	HCC	310	222	HB	PCR-RFLP	Yes	NR
24	Okubo et al., [18] ^@^	2010	Japan	Asian	GC	552	697	HB	PCR-RFLP	Yes	NR
25	Chen et al., [67]	2011	China	Asian	CRC	126	407	HB	PCR-LDR	Yes	age/sex
26	Yue et al., [68]	2011	China	Asian	CC	447	443	HB	PCR-RFLP	Yes	age
27	Mittal et al., [69]	2011	India	Caucasian	UBC	212	250	HB	PCR-RFLP	Yes	age/sex
28	Zhou et al., [70]	2011	China	Asian	CC	226	309	HB	PCR-RFLP	Yes	age
29	Akkiz et al., [71]	2011	Turkey	Caucasian	HCC	185	185	HB	PCR-RFLP	Yes	age/sex/smoking/alcohol
30	Zhan et al., [72]	2011	China	Asian	CRC	252	543	HB	PCR-RFLP	Yes	age/sex
31	Hong et al., [73]	2011	Korea	Asian	NSCLC	406	428	PB	TaqMan	Yes	age/sex
32	Permuth-Wey et al., [74]	2011	USA	Caucasian	Glioma	593	614	PB	Illumina’s GoldenGate	Yes	NR
33	Akkiz et al., [75]	2011	Turkey	Caucasian	HCC	222	222	HB	PCR-RFLP	Yes	age/sex/smoking/alcohol
34	Zhu et al., [76]	2011	China	Asian	CRC	573	588	HB	TaqMan	Yes	age/sex
35	Zhou et al., [25]	2011	China	Asian	HCC	186	483	HB	PCR-RFLP	Yes	NR
36	Schuetz et al., [77]	2012	Canada	Caucasian	NHL	717	694	PB	Illumina’s GoldenGate	Yes	age/sex/area
37	Xiang et al., [26]	2012	China	Asian	HCC	100	100	HB	PCR-RFLP	Yes	NR
38	Jedlinski et al., [30]	2011	Australia	Caucasian	BC	193	190	PB	PCR-RFLP	Yes	age
39	Yang et al., [21] *	2008	USA	Caucasian	UBC	746	746	HB	SNPlex	Yes	age/sex
40	George et al., [19] !	2011	India	Caucasian	PC	159	230	HB	PCR-RFLP	Yes	age/sex
41	Wang et al., [78]	2010	China	Asian	ESCC	458	489	PB	SNaPshot	Yes	age/sex/area
42	Zhang et al., [79]	2011	China	Asian	HCC	302	513	HB	PCR-RFLP	Yes	NR
43	Zhang et al., [80]	2012	China	Asian	BC	252	248	PB	PCR-RFLP	Yes	age/sex/area
44	Pastrello et al., [27]	2010	Italy	Caucasian	BC/OC	101	155	NR	Sequencing	Yes	NR
45	Vinci et al., [28]	2011	Italy	Caucasian	NSCLC	101	129	NR	HRMA	Yes	age/sex
46	Zhou et al., [81]	2012	China	Asian	GC	1686	1895	HB	TaqMan	Yes	age/sex
47	Kim et al., [29]	2012	Korea	Asian	HCC	159	201	PB	PCR-RFLP	Yes	NR
48	Smith et al., [82]	2012	Australia	Caucasian	BC	193	193	HB	HRMA	Yes	age/sex/ethnicity
49	Hishida et al., [83]	2011	Japan	Asian	GC	583	540	HB	PCR-CTPP	Yes	age/sex
50	Horikawa et al., [84]	2008	USA	Caucasian	RCC	279	278	PB	SNPlex	Yes	age/sex/ethnicity/residence
51	Lung et al., [85]	2012	China	Asian	NPC	233	3786	PB	Tm-shift	Yes	age/sex
52	Chu et al., [20] &	2012	Taiwan	Asian	OSCC	470	425	HB	PCR-RFLP	Yes	NR
53	Bae et al., [86]	2012	Korea	Asian	HCC	417	404	HB	TaqMan	Yes	NR

HCC: hepatocellular cancer; BC: breast cancer; GBC: gallbladder cancer; GC: gastric cancer; NSCLC: non-small cell lung carcinoma; CC: cervical cancer; LC: lung cancer; EC: esophageal cancer; PC: prostate cancer; HNSCC: head and neck squamous cell carcinoma; NHL: Non-Hodgkin lymphoma; OC: ovarian cancer; PTC: papillary thyroid carcinoma; NSCLC: non-small cell lung cancer; RCC: renal cell carcinoma; UBC: urinary bladder cancer; CRC: colorectal cancer; ESCC: esophageal squamous cell carcinoma; NPC: Nasopharyngeal Carcinoma; OSCC: oral squamous cell carcinoma; HWE: Hardy-Weinberg equilibrium; PCR-RFLP: polymerase chain reaction-restriction fragment length polymorphism; PCR–LDR: polymerase chain reaction–ligation detection reaction; PCR-FRET: polymerase chain reaction–fluorescent resonance energy transfer; HRMA: high-resolution melting analysis; PCR-CTPP: polymerase chain reaction with confronting two-pair primers; Tm-shift: Melting-temperature–shift allele-specific genotyping; HB: hospital based; PB: population based; NR: not reported;

* Let7f-2 rs17276588 deviated from HWE in controls.

$ mir-492 rs2289030 and mir-149 rs2292832 deviated from HWE in controls.

# Cirrhosis patients without HCC served as controls.

@ miR-499 rs3746444 deviated from HWE in controls.

! mir196a2 rs11614913 and mir146a rs2910164 deviated from HWE in controls.

& miRNA149 rs2292832 deviated from HWE in controls.

## Methods

### Publication Search

We searched the PubMed, Medline and Embase databases using the search terms “miRNA,” “cancer/carcinoma,” and “polymorphism/variant” updated until August 25, 2012 and limited to English language papers. Identification of meta-analyses of association studies on miRNA polymorphisms and cancer was also carried out through a search of electronic databases of PubMed, Medline and Embase, up to August 2012. The Medical Subject Headings and key words used for the search were “miRNA”, “cancer”, “polymorphism”, and “meta-analysis” (with both synonymous and plural forms). The online searching was accompanied by checking reference lists from the identified articles and reviews for potentially eligible original reports.

### Inclusion and Exclusion Criteria

All miRNA association studies were included in the present meta-analysis if they met the following criteria: 1) case-control study, 2) outcome cancer (histologically/pathologically proven), and 3) sufficient data for examining an odds ratio (OR) with 95% confidence interval (95% CI). The major exclusion criteria were as follows: 1) duplicate data, 2) case reports, series, abstract, comment, review and editorial and 3) insufficient data. Articles published in a language other than English were also excluded.

### Data Extraction

From each study, information like: author, year of publication, country of origin, cancer type, ethnicity, number of cases and controls, source of control groups (study design) and genotyping method was extracted. In some cases, identical data were described in more than one publication; in such cases the secondary studies were not included in the meta-analysis. In a few studies, part of the data had already been reported elsewhere, therefore, only the novel data was included. We also checked for HWE in control subjects among all publications.

### Genotype and Allele Distributions

Genotype distributions were extracted from the eligible publications for each polymorphism or computed from allele frequencies (if genotype frequencies were not reported) on the basis of sample size, assuming Hardy-Weinberg equilibrium (HWE).

### Methodological Quality Assessment

The quality of selected studies was evaluated by scoring according to a set of predetermined criteria. The categories in scoring system used for assessing study quality are summarized in Table S1 [11]. Quality scores ranged from 0 to 10 and studies were scored as “good” if the score was 8–10, “fair” if the score was 5–7 and “poor” if the score was <4.

### Statistical Analysis

In the present meta-analysis, we investigated the potential association between the variant allele of miRNA polymorphisms and cancer risk. Also, analysis between the heterozygote, the homozygote and also in dominant and recessive models was done to estimate cancer risk. Stratified analyses were performed by tumor site, ethnicity and source of controls (hospital or population based). Other potentially relevant sub-group analyses such as age, sex and cancer subgroup could not reliably be investigated due to limited data availability. Between-study heterogeneity was evaluated with a χ^2^-based Q-test among the studies [12]. Heterogeneity was considered significant when P<0.05. In case of no significant heterogeneity, point estimates and 95% CI was estimated using the fixed effect model (Mantel–Haenszel), otherwise, random effects model (DerSimonian Laird) was employed [13], [14]. The significance of overall odds ratio (OR) was determined by the Z-test. A χ^2^ test with one degree of freedom was performed in controls to observe deviation from HWE. Publication bias was weighted by Begg’s funnel plot and Egger’s linear regression method with P<0.05 being considered statistically significant [15]. To assess the stability of the results, sensitivity analyses were performed. Each study in turn was removed from the total, and the remaining studies were reanalyzed. Moreover, sensitivity analysis was also performed, excluding studies whose allele frequencies in controls exhibited significant deviation from the HWE, given that the deviation may denote bias [16]. The type I error rate was fixed at 0.05. All the p values were two sided and all the statistical tests were implemented using the Comprehensive Meta-analysis software (Version 2.0, BIOSTAT, Englewood, NJ).

### Hardy–Weinberg Equilibrium Correction

For evaluating impact of HWE-deviated studies on point estimates in genotype based contrasts, ORs were corrected by using the HWE-predicted genotype count in controls instead of the observed counts, as recommended by Trikalinos et al. [17]; thereafter, they were incorporated in the sensitivity analysis.

## Results

### Study Characteristics

Table 1 and Table S2 show the characteristics of eligible studies and genotype frequency distributions of studied miRNA SNPs included in the present meta-analysis. Fifty-three studies published between 2008 and August 2012 met our inclusion criteria with a total of 27573 cancer cases and 34791 controls. Two studies in Chinese language and 12 cohort studies were excluded from the present analysis. The total score of most studies was over 7 (Table S3). Thirty-two of the studies were conducted on subjects with Asian ethnicity (14689 cases/19894 controls) and 21 with Caucasian ethnicity (12884 cases/14897 controls). Malignances were histologically or pathologically confirmed in 35 of the included studies, while in 11 studies it was not defined. Controls in 19 studies were population-based, while controls of 31 studies were hospital-based. Two studies included both population-based and hospital-based controls while another 2 studies did not report about the control source. Twelve out of 53 studies did not report on the matching criteria for controls while other studies recruited controls corresponding to cases by the age/sex/area. A classical polymerase chain reaction–restriction fragment length-polymorphism (PCR–RFLP) method was adopted in 28 of the 53 studies. Seven studies used TaqMan assay; four studies used direct sequencing of the polymorphism; three studies used SNPlex; two studies used SNaPshot, Illumina’s GoldenGate, high-resolution melting analysis (HRMA) and polymerase chain reaction–ligation detection reaction (PCR-LDR) assay each while 1 study each used fluorescence labeled hybridization (PCR-FRET), MALDI-TOF mass spectrometry, polymerase chain reaction with confronting two-pair primers (PCR-CTTP), Melting-temperature–shift allele-specific genotyping (Tm-shift) and Sequenom’s MassARRAY as genotyping methods. The distributions of studied SNPs genotype in all the studies were in accordance with HWE in the control cohort, except for five studies [18]–[23] (Table 1 footnote).

### Quantitative Data Synthesis

#### miR-146a rs2910164

The miR-146a rs2910164 polymorphism was analyzed in 27 studies with 12088 cases and 17340 controls and was not found to be associated with cancer risk (OR = 0.918, 95% CI = 0.777–1.086, *P*-value = 0.320; CC vs. GG; Table 2). Significant heterogeneity was observed (*Q* = 77.126, *P* = <0.001, *I^2^* = 66.289%, CC vs. GG). Similar results were obtained at the allelic level and for other genetic models also with significant heterogeneity (P_Het_ = <0.001–0.008; Table 2). After the exclusion of the study by George et al. [19], whose genotypic distribution in controls deviated from HWE, the results did not significantly alter from the corresponding pooled OR (OR = 0.919, 95% CI = 0.775–1.090, *P*-value = 0.331; CC vs. GG). Removing 8 low score studies {[18], [19], [24]–[29] with score ≤5}, did not altered the pooled results (Table S4a).

**Table 2:** Meta-analysis of mir-146a rs2910164 polymorphism.

Variables	n^a^	Cases/Controls	C-allele vs. G-allele		CC vs. GG		CG vs. GG		Dominant(CC+CG vs. GG)		Recessive(CC vs. CG+GG)	
			OR (95% CI)	*P_Het_*	OR(95% CI)	*P_Het_*	OR(95% CI)	*P_Het_*	OR(95% CI)	*P_Het_*	OR(95% CI)	*P_Het_*
Total	27	12088/17340	0.963(0.892–1.039)	<0.001	0.918(0.777–1.086)	<0.001	1.008(0.929–1.094)	0.008	1.002(0.919–1.092)	<0.001	1.034(0.894–1.196)	<0.001
**Cancer type**												
Hepatocellular	5	1146/1510	1.102(0.981–1.237)	0.242	0.764(0.590–0.988)	0.313	1.087(0.899–1.314)	0.284	1.126(0.940–1.349)	0.229	1.148(0.939–1.403)	0.293
Breast	3	2669/3395	1.023(0.945–1.107)	0.938	1.098(0.912–1.322)	0.539	0.989(0.887–1.103)	0.888	1.007(0.908–1.118)	0.955	1.094(0.920–1.300)	0.453
Other	19	8273/12435	0.934(0.844–1.035)	<0.001	0.913(0.727–1.146)	<0.001	0.999(0.896–1.115)	0.002	0.985(0.877–1.106)	<0.001	1.009(0.826–1.232)	<0.001
**Ethnicity**												
Asian	14	5914/10118	0.889(0.785–1.007)	<0.001	0.818(0.650–1.029)	<0.001	0.972(0.891–1.060)	0.084	0.931(0.800–1.083)	<0.001	0.925(0.770–1.112)	<0.001
Caucasian	13	6174/7222	1.050(0.993–1.111)	0.459	1.102(0.886–1.370)	<0.001	1.046(0.974–1.124)	0.017	1.055(0.985–1.130)	0.114	1.274(1.096–1.481)	0.153
**Study design**												
Population based	9*	5706/10226	1.015(0.898–1.147)	0.379	1.185(0.936–1.500)	0.008	1.070(0.990–1.156)	0.051	1.089(1.012–1.172)	0.265	1.331(1.076–1.645)	0.002
Hospital based	17*	6409/10606	0.893(0.799–0.997)	<0.001	0.809(0.650–1.007)	<0.001	0.950(0.878–1.029)	0.117	0.922(0.813–1.044)	0.002	0.922(0.762–1.115)	<0.001

Random effects model was used when *P* value of Q for heterogeneity test (*P_Het_*)<0.05; otherwise, fixed effect model was used.

a Number of studies involved.

* The study by Lung et al., [85] has both hospital based and population based controls.

OR: odds ratio; CI: confidence interval.

Stratified analyses significantly reduced the heterogeneity of the subgroups. Based on different cancer types, non-significant increased risk was found in hepatocellular cancer (OR = 1.1–1.2; Table 2) and borderline increased risk for breast cancer (OR∼1.0–1.1). However, no significant association was found in other cancers (including lung, gastric, cervical, esophageal, gallbladder, urinary bladder, prostate, head and neck, thyroid and glioma).

In the stratified analysis based on ethnicity of study population, there was a strong association between rs2910164 and overall cancer risk in Caucasian population under recessive model (OR = 1.274, 95%CI = 1.096–1.481, *P* = 0.002; *I^2^* = 28.99%). However, this association was lost in Asian populations (Table 2). Further subgroup analysis demonstrated that the rs2910164 ‘C’ allele was associated with significantly increased cancer risk in population based study design (OR = 1.1–1.3) (Table 2).

#### mir-196a2 rs11614913

Thirty studies investigated mir-196a2 rs11614913 polymorphism and its association with cancer (13703 cases and 15439 controls). The “T” allele of the polymorphism was considered as the variant allele in the present analysis. The overall OR showed statistically significant association between the rs11614913 polymorphism and reduced risk of cancer (OR = 0.846, 95% CI = 0.747–0.958, *P_Het_*<0.001: TT vs. CC and OR = 0.941, 95% CI = 0.889–0.996, *P_Het_*<0.001: T vs. C allele; Table 3). After the exclusion of the study by George et al. [19], whose genotypic distribution in controls deviated from HWE, the results did not significantly altered from the corresponding pooled OR (OR = 0.849, 95% CI = 0.749–0.962, *P*-value = 0.010: TT vs. CC). Removing low scoring studies did not make significant deviation from the above obtained results {[18], [19], [28]–[30] (Table S4b)}.

**Table 3:** Meta-analysis of mir-196a2 rs11614913 polymorphism.

Variables	n^a^	Cases/Controls	T-allele vs. C-allele		TT vs. CC		TC vs. CC		Dominant(TT+TC vs. CC)		Recessive(TT vs. TC+CC)	
			OR (95% CI)	*P_Het_*	OR(95% CI)	*P_Het_*	OR(95% CI)	*P_Het_*	OR(95% CI)	*P_Het_*	OR(95% CI)	*P_Het_*
Total	30	13703/15439	0.941(0.889–0.996)	<0.001	0.846(0.747–0.958)	<0.001	1.017(0.934–1.106)	0.001	0.972(0.890–1.060)	<0.001	0.854(0.778–0.939)	<0.001
**Cancer type**												
Breast	5	3449/4140	0.914(0.804–1.040)	0.020	0.812(0.607–1.085)	0.014	0.939(0.850–1.037)	0.532	0.911(0.829–1.000)	0.148	0.872(0.703–1.080)	0.027
Lung	4	2219/2232	0.893(0.821–0.971)	0.149	0.793(0.671–0.938)	0.259	0.927(0.801–1.074)	0.059	0.882(0.768–1.013)	0.075	0.842(0.737–0.962)	0.201
Colorectal	3	951/1538	0.848(0.754–0.954)	0.223	0.690(0.543–0.876)	0.150	0.886(0.719–1.091)	0.636	0.813(0.667–0.990)	0.351	0.751(0.621–0.909)	0.209
Hepatocellular	4	1015/999	0.862(0.683–1.088)	0.019	0.744(0.466–1.189)	0.022	0.897(0.721–1.115)	0.631	0.850(0.692–1.043)	0.190	0.809(0.567–1.154)	0.037
Other	14	6069/6530	0.994(0.912–1.083)	0.001	0.915(0.744–1.124)	<0.001	1.125(0.975–1.298)	0.001	1.080(0.941–1.240)	<0.001	0.873(0.740–1.029)	<0.001
**Ethnicity**												
Asian	17	7718/8580	0.905(0.845–0.969)	0.004	0.820(0.699–0.963)	<0.001	0.983(0.866–1.116)	0.003	0.923(0.814–1.047)	0.001	0.831(0.748–0.923)	0.006
Caucasian	13	5985/6859	0.994(0.908–1.088)	0.002	0.889(0.729–1.084)	0.002	1.056(0.944–1.183)	0.039	1.033(0.918–1.162)	0.010	0.889(0.741–1.067)	0.002
**Study design**												
Population based	12*	6520/7355	0.901(0.833–0.975)	0.010	0.777(0.651–0.928)	0.003	0.946(0.877–1.021)	0.262	0.905(0.842–0.972)	0.136	0.812(0.693–0.952)	0.001
Hospital based	18*	7508/8421	0.945(0.873–1.022)	<0.001	0.845(0.708–1.009)	<0.001	1.032(0.910–1.170)	<0.001	0.984(0.863–1.121)	<0.001	0.854(0.755–0.965)	0.002

Random effects model was used when *P* value of Q-test for heterogeneity test (*P_Het_*)<0.05; otherwise, fixed effect model was used.

a Number of studies involved.

* The study by Hoffman et al., [22] has both hospital based and population based controls.

OR: odds ratio; CI: confidence interval.

Stratified analysis by cancer type showed that this association was significant in lung and colorectal cancer at the allelic and genotypic level (except TC vs. CC) (Table 3). However, the association was lost under the dominant model in lung cancer. No significant association was found in other cancers (including gastric, cervical, esophageal, gallbladder, urinary bladder, prostate, head and neck and glioma).

In the stratified analysis by ethnicity, Asian individuals had lower risk of cancer under both the allelic and genotypic level (OR<1.0), whereas Caucasian individuals did not show any significant association under any genetic model. Additional subgroup analysis significantly associated rs11614913 ‘T’ allele with decreased cancer risk in population based study design (OR = 0.77–0.90) (Table 3).

#### mir-499 rs3746444

Fourteen studies evaluated mir-499 rs3746444 polymorphism and its association with cancer. There was a marginally increased overall risk of cancer under the allelic and genotypic models [OR = 1.130, 95%CI = 1.002–1.275, *P* = 0.046, *I* ^2^ = 72.85% (C vs. T allele) and OR = 1.177, 95%CI = 1.007–1.377, *P* = 0.041, *I* ^2^ = 74.66% (CT vs. TT); Table 4]. After the exclusion of the study by George et al. [19], whose genotypic distribution in controls deviated from HWE, the borderline significant association was lost (*P* = 0.066; allelic model). However, no association was found between genotype CC and cancer risk under the other models. Based on the ethnicity of study population, association was found in Asian populations under allelic and recessive models (Table 4). Removing low scoring studies did not alter the above obtained results {[18], [19], [25], [26], [28], [29] (Table S4c)}.

**Table 4:** Meta-analysis of mir-499 rs3746444 polymorphism.

Variables	n^a^	Cases/Controls	C-allele vs. T-allele		CC vs. TT		CT vs. TT		Dominant(CC+CT vs. TT)		Recessive(CC vs. CT+TT)	
			OR (95% CI)	*P_Het_*	OR(95% CI)	*P_Het_*	OR(95% CI)	*P_Het_*	OR(95% CI)	*P_Het_*	OR(95% CI)	*P_Het_*
Total	14	7141/8479	1.130(1.002–1.275)	<0.001	1.124(0.964–1.310)	0.055	1.177(1.007–1.377)	<0.001	1.141(0.985–1.322)	<0.001	1.091(0.871–1.368)	0.035
**Cancer type**												
Breast	2	2588/3260	1.115(0.878–1.417)	0.017	1.257(0.701–2.255)	0.036	1.067(0.952–1.196)	0.163	1.079(0.967–1.203)	0.056	1.111(0.869–1.421)	0.050
Hepatocellular	3	436/784	1.134(0.641–2.006)	0.001	1.245(0.357–4.338)	0.023	1.001(0.762–1.314)	0.074	1.116(0.625–1.993)	0.009	1.515(0.839–2.734)	0.062
Other	9	4117/4435	1.139(0.976–1.330)	<0.001	1.074(0.875–1.320)	0.383	1.262(0.992–1.606)	<0.001	1.175(0.948–1.456)	<0.001	1.012(0.828–1.238)	0.130
**Ethnicity**												
Asian	8	3751/4343	1.227(1.006–1.497)	<0.001	1.402(0.941–2.088)	0.037	1.210(0.972–1.506)	<0.001	1.243(0.994–1.554)	<0.001	1.357(1.062–1.734)	0.074
Caucasian	6	3390/4136	0.989(0.916–1.067)	0.343	0.976(0.803–1.186)	0.875	1.140(0.895–1.451)	0.001	0.967(0.878–1.064)	0.237	0.936(0.773–1.132)	0.394
**Study design**												
Population based	5	4026/4726	1.037(0.961–1.119)	0.021	1.098(0.886–1.360)	0.126	1.027(0.935–1.128)	0.217	1.036(0.947–1.133)	0.065	1.086(0.879–1.342)	0.200
Hospital based	8	3014/3624	1.206(0.926–1.570)	<0.001	1.246(0.874–1.776)	0.045	1.369(1.017–1.844)	<0.001	1.360(1.033–1.789)	<0.001	1.113(0.763–1.624)	0.016

Random effects model was used when *P* value of Q-test for heterogeneity test (*P_Het_*)<0.05; otherwise, fixed effect model was used.

a Number of studies involved.

OR: odds ratio; CI: confidence interval.

#### mir-149 rs2292832

Seven studies evaluated mir-149 rs2292832 and its association with cancer risk. The results of the overall meta-analysis did not suggest any association between rs2292832 and cancer susceptibility for all genetic models (Table 5). Exclusion of the study by Vinci et al. [31] and Kim et al., [29] with quality score of 2 did not altered the pooled estimate (Table S4d).

**Table 5:** Meta-analysis of mir-149 rs2292832 polymorphism.

Variables	n^a^	Cases/Controls	T-allele vs. C-allele		TT vs. CC		TC vs. CC		Dominant(TT+TC vs. CC)		Recessive(TT vs. TC+CC)	
			OR (95% CI)	*P_Het_*	OR(95% CI)	*P_Het_*	OR(95% CI)	*P_Het_*	OR(95% CI)	*P_Het_*	OR(95% CI)	*P_Het_*
Total	7	4142/4242	0.994(0.924–1.069)	0.345	1.000(0.859–1.165)	0.324	0.982(0.893–1.081)	0.526	0.988(0.902–1.083)	0.351	1.041(0.913–1.187)	0.333
**Cancer type**												
Breast	2	1254/1322	0.949(0.845–1.067)	0.167	0.931(0.709–1.222)	0.418	0.919(0.781–1.082)	0.106	0.921(0.789–1.077)	0.101	0.971(0.750–1.259)	0.737
Other	5	2888/2920	1.018(0.938–1.103)	0.417	1.034(0.860–1.243)	0.205	1.018(0.904–1.146)	0.816	1.025(0.916–1.147)	0.591	1.066(0.915–1.241)	0.172
**Ethnicity**												
Asian	5	2932/2983	0.991(0.916–1.073)	0.418	1.003(0.838–1.200)	0.486	0.968(0.860–1.088)	0.314	0.978(0.875–1.094)	0.240	1.056(0.910–1.225)	0.499
Caucasian	2	1210/1259	1.004(0.887–1.136)	0.094	0.993(0.742–1.328)	0.061	1.013(0.858–1.197)	0.658	1.009(0.861–1.182)	0.297	0.988(0.746–1.310)	0.068
**Study design**												
Population based	4	2562/2558	0.996(0.908–1.094)	0.324	0.980(0.815–1.179)	0.496	0.979(0.868–1.103)	0.275	0.990(0.883–1.110)	0.206	1.088(0.917–1.291)	0.409
Hospital based	2	1579/1555	0.957(0.853–1.074)	0.688	1.016(0.670–1.539)	0.234	0.976(0.826–1.154)	0.323	0.960(0.819–1.124)	0.363	0.930(0.753–1.149)	0.742

Random effects model was used when *P* value of Q-test for heterogeneity test (*P_Het_*)<0.05; otherwise, fixed effect model was used.

a Number of studies involved.

OR: odds ratio; CI: confidence interval.

Through stratified analyses, no significant associations were found in any of the subgroups (racial descent, cancer types and study design) (Table 5).

The effect of some polymorphisms could not be evaluated due to the limited number of studies (mir-27a rs895819 and mir-373 rs12983273 and rs10425222 = 3 studies; mir-100 rs1834306, mir-124-1 rs531564, mir-128 rs11134527, mir-155 rs928883 and rs2829803, mir-15a rs9535416 and rs2476391, mir-1792 rs17642969, mir-219 rs107822 and rs213210, mir-26a1 rs7372209, mir-30a rs1358379, mir-30c1 rs16827546, mir-335 rs3807348 and rs41272366, mir-423 rs6505162, mir-492 rs2289030, mir-604 rs2368392, mir-608 rs4919510 and mir-631 rs5745925 = 2 studies; mir-618 rs2682818, mir-605 rs2043556, mir-34b/c rs4938723, mir-126 rs4636297, let7f-2 rs17276588, let-7a3 rs731085, mir-101-1 rs7536540, mir101-2 rs17803780 and rs12375841 and mir-338 rs62073058 = 1 study each).

### Sensitivity Analysis

A single study involved in the meta-analysis was removed each time to reflect the influence of the individual data set to the pooled ORs for each of the studied miRNA polymorphisms. The corresponding pooled ORs were not significantly altered for any of the SNPs studied (Table S5a-d).

### Publication Bias Analysis

Publication bias was assessed by performing funnel plot and Egger's regression test under all models. For mir-149 rs2292832, because the number of included studies was small, we did not perform publication bias analysis. After combining all the cancer types, a little asymmetry was observed for mir-146a rs2910164, but the results of Egger's regression test suggested no evidence for publication bias (Y axle intercept = -0.896, (95% CI) = −3.047 to 1.253; t = 0.859, *p* = 0.398 for allelic model) (Figure S1). Also, Begg and Mazumdar rank correlation test indicated absence of publication bias (*P*_2tailed_ = 0.646). Similarly for mir-196a2 rs11614913 and mir-499 rs3746444, funnel plots were symmetrical and the Egger's test for both models showed no significance, suggesting little evidence of publication bias (Figure S2 and S3).

A cumulative meta-analysis was also done by sorting the studies in the sequence of largest to smallest, and analysis performed with the addition of each study. The point estimate of the study did not deviate with the addition of smaller studies, ruling out the possibility of publication bias for all the analyzed miRNA SNPs.

### Meta-analyses of Association Studies on miRNA SNPs

Eleven meta-analyses published in 2011 and 2012 were retrieved, focusing on 2 miRNA polymorphisms (miR-146a rs2910164 and miR-196a2 rs11614913). Table 6 shows the main characteristics of individual meta-analyses included. The number of primary studies included in the meta-analyses ranged from 4 to 27 with the number of subjects included spanning from 3007 to 10569. The results of the published meta-analyses of the association between miRNA SNPs and cancer showed an overall statistically significant increased risk for mir-196a2 rs11614913 (variant C allele). In subgroup analysis, the increased risk was more prominent in digestive system cancers such as breast, colorectal and hepatocellular cancer. For mir-146a rs2910164, in an overall analysis, no significant associations were found. However, in the stratified analysis, this polymorphism was associated with increased breast cancer risk among Europeans [32] and negatively associated with digestive system cancer [33]. The results are also consistent with the outcome from our present meta-analysis.

**Table 6:** Description of meta-analyses included in the systematic review.

S. no.	Reference	PublicationYear	Cancer Type	Cases/controls	miRNA	rs number	P_het_ *	OR*	95% CI*
1	Lian et al., [32]	2012	BC	4238/4469	miR-146a	rs2910164	0.757	1.16	0.98–1.36
2	Guo et al., [11]	2012	HCC, CRC, GBC, PSCC, OSCC, ESCC, GC	4999/7606	miR-196a2	rs11614913	0.0003	1.38	1.13–1.67
3	Xu et al., [41] *	2011	BC, LC, PTC, HCC, GBC, HNSCC, PC, ESCC	7183/7943	miR-146a	rs2910164	0.03	0.89	0.75–1.05
			BC, LC, GBC, Glioma, HNSCC, GC	7992/8849	miR-196a2	rs11614913	0.45	0.92	0.85–0.99
4	Wang et al., [87]	2012	LC, BC, GC, Glioma, GBC, HNSCC	6540/7562	miR-196a2	rs11614913	0.021	1.18	1.01–1.39
5	Chu et al., [88]	2011	BC, ESCC, LC, GC, HCC, GBC, PC, HNSCC, Glioma	9341/10569	miR-196a2	rs11614913	<0.001	1.22	1.04–1.44
6	Gao et al., [89]	2011	BC	3007/3718	miR-146a	rs2910164	0.65	0.90	0.75–1.07
				3287/4298	miR-196a2	rs11614913	0.03	1.30	1.01–1.68
7	Qiu et al., [90]	2011	BC, UBC, ESCC, OC, CCGBC, GC, HCC, LC, PTC, HNSCC, Glioma	10585/12183	miR-146a	rs2910164	<0.001	1.13	0.93–1.37
8	Qiu et al., [91]	2011	BC, UBC, ESCC, OC, CCGBC, GC, HCC, LC, PTC, HNSCC, Glioma, CRC	10441/12353	miR-196a2	rs11614913	<0.001	1.30	1.14–1.48
9	Zhang et al., [92]	2012	LC, HCC, BC, CRC, GC, ESCC, GC, Glioma, UBC, PC	10435/12075	miR-196a2	rs11614913	<0.001	1.23	1.08–1.39
10	Wang et al., [93]	2012	BC, GC, PC, UBC, CC, ESCC, OC, HCC, PTC, RCC, GBC	10496/12885	miR-146a	rs2910164	0.09	1.16	0.98–1.38
11	Wang et al., [94]	2012	LC, GC, CRC, GBC, HCC, ESCC	2394/2767	miR-146a	rs2910164	0.02	1.17	0.95–1.44

BC: breast cancer; GBC: gallbladder cancer; GC: gastric cancer; LC: lung cancer; PC: prostate cancer; HNSCC: head and neck squamous cell carcinoma; PTC: papillary thyroid carcinoma; ESCC: esophageal squamous cell carcinoma; HCC: hepatocellular cancer; CRC: colorectal cancer; OSCC: oral squamous cell carcinoma; PSCC: pharynx squamous cancer; OC: ovarian cancer; CC: cervical cancer; RCC: renal cell cancer.

* Considered T allele as variant allele as in the present study.

P_het_, *p*-value for heterogeneity.

OR, odds ratio.

CI, confidence interval.

* Homozygous wild vs. homozygous variant genotype.

We also computed the population-attributable risk (PAR) to refer to the proportion of disease risk in Caucasians and Asians that can be attributed to the causal effects of the risk SNP (variant genotype). PAR can be assessed by using the formula [34]: PAR (%) = (OR-1)/OR × (number of exposed cases/total number of cases) × 100%, where OR is the pooled OR stratified for ethnicity derived from the meta-analyses incorporating the largest number of individuals. The results showed mir-196a2 rs11614913 to be the most impacting polymorphism (which might account for approximately 15% among Asians) [PAR (%) mir-196a2 rs11614913 ‘T’ allele carriers: Asians = 14.9, Caucasians = 1.4]. Although the ORs and allele frequencies used for computing PAR were taken from the same ethnic group, the results could still be biased due to the difference in geographic areas and population stratification in individual studies. A more consistent estimation of the PAR requires additional statistics to identify population subgroups significantly affected by particular miRNA polymorphism.

## Discussion

In the present study, we reviewed the available literature on genetic studies of miRNA SNPs in cancer and conducted four independent meta-analyses for association between overall cancer and mir-146a rs2910164, mir-196a2 rs11614913, mir-149 rs2292832 and mir-499 rs3746444 polymorphisms. Our results associated mir-196a2 rs11614913 with a decreased overall cancer risk. Meanwhile, there was no association between other studied miRNA SNPs. However, due to the small number of studies with an overall mediocre quality and lack of confirmatory studies, it is very difficult to draw any definitive conclusions.

miR-146a, first found in mouse, has been shown to play an important role in tumorigenesis by promoting cell proliferation and colony formation in NIH/3T3 cells [35]–[37]. It has also been shown to play an important role in suppressing metastatic ability in breast cancer, prostate cancer and MDA-MB-231 cells [38]–[40]. A ‘G’ to ‘C’ substitution (rs2910164) located in the middle of the stem hairpin on the passenger strand of the precursor of miR-146a has a lower transcriptional activity due to decreased nuclear pri-miR-146a processing efficiency leading to low levels of mature miR-146a in cells with homozygous variant genotype (CC) [24]. Also, the change decreases free energy (dG) from -42.40 kcal/mol for G allele to -39.60 kcal/mol for C allele, signifying a less stable secondary structure for the C allele compared with the G allele (Table S6). No significant association between this polymorphism and overall cancer risk was found in our meta-analysis replicating a previous meta-analysis study [33]. However, the variation was associated with increased cancer risk in Caucasians and studies with population based design. This could be explained on the fact that most of the population based studies were in Caucasian population.

Aberrant mir-196a2 expression is implicated in cancer susceptibility and metastasis in several malignancies [41], [42]. Human miR-196a2 comprises two different mature miRNAs (miR-196a and miR196a*) processed from same stem-loop. The rs11614913 polymorphism lies in the mature sequence of miR-196a* and negatively impacts endogenous processing of either miRNA precursor to its mature form [43] and is associated with various malignancies [42], [44]. The rs11614913 ‘C’ allele increases the expression levels of mature hsa-mir-196a2 compared to ‘T’ allele and the SNP also affects the binding of mature hsa-miR-196a2 to its target mRNA [44].We observed that there was a significantly decreased risk of overall cancer with this polymorphism at allelic and recessive level as with previous studies [11], [33], [45], [46]. When stratified by cancer types, the association was found in lung and colorectal cancer only which might be caused by the different microenvironments and mechanisms in different cancer types.

The mir-499 microRNA has also been implicated in several human malignancies (Table S1). A T>C (rs3746444) polymorphism has been identified in the stem region of the mir-499 gene resulting in A:U to G:U mismatch in the stem structure of miR-499 precursor. This SNP has been shown to be associated with risk for various cancers as evident from association studies (Table S1), however the mechanism remains unknown. This polymorphism increased the risk of cancer in the dominant genetic model. The association was significant with hepatocellular cancer in Asians, which demonstrates that Asian populations with this polymorphism might be more susceptible to hepatocellular cancer compared to Europeans. Moreover, the population attributable risk (PAR) for this polymorphism was also around 15% among Asians, signifying its importance.

For mir-149 rs2292832, no statistical association was found in the overall comparison and subgroup analysis. Because of the limited number of studies (7) for this polymorphism, the results should be interpreted with caution.

For other miRNA polymorphisms, because of limited number of studies (ranging from one to three), meta-analysis was not done as it would not have been reliable.

One of the important concerns in every meta-analysis is publication bias. Because meta-analysis reviews quantitative data from numerous studies, the publication bias effect of the literature incorporated in the study can bias the meta-analytic outcome. In the present study, the funnel plot for overall results was symmetrical for all the analyzed miRNA SNPs, indicating negligible likelihood of publication bias. The Egger’s test and Begg and Mazumdar rank correlation test were also negative for publication bias. However, the possibility of publication bias cannot completely be ruled out [47]. Sensitivity analyses using HWE-adjusted ORs and corresponding variances also did not modify the results.

To the best of our knowledge, the present study is the most comprehensive meta-analysis to date to have assessed the relationship between the miRNA polymorphisms and cancer risk. Nevertheless, our meta-analysis had some limitations common to these types of studies. First, the present meta-analysis only included case-control studies, most of which were hospital based and excluded 12 cohort studies to avoid potential heterogeneity in comparing results. Thus, the controls may not reflect the representative element of the source population. Second, the difference in the geographic areas (environmental factors) and genetic backgrounds of the study cohort in each article could influence the results. Third, the low sample size in some of the included studies might influence the statistical power to better evaluate the association between miRNA polymorphisms and overall cancer, especially in subgroup analysis. Fourth, gene-gene and gene-environment interactions were not analyzed which might alter the associations between miRNA gene polymorphisms and cancer. Also, a more precise analysis stratified by variables such as age, sex etc. could not be performed due to limitations of the data which also restricted our ability to detect possible sources of heterogeneity.

In conclusion, the results of our meta-analysis demonstrate that mir-196a2 rs11614913 polymorphisms have significant associations with overall cancer risk, although some results are limited by the small number of studies. However, no significant association exists between mir-146a rs2910164, mir-499 rs3746444 and mir-149 rs2292832 and overall cancer. Further studies with a large sample size are needed to evaluate their association with cancer risk.

## Supporting Information

Figure S1 **Begg’s funnel plot of publication bias for miR-146a rs2910164.** Log OR is plotted versus standard error of Log OR for each included study. Every circle dot represents a separate study for the indicated association (C versus G).
(TIF)

Figure S2 **Begg’s funnel plot of publication bias for mir-196a2 rs11614913.** Log OR is plotted versus standard error of Log OR for each included study. Every circle dot represents a separate study for the indicated association (TT versus CC).
(TIF)

Figure S3 **Begg’s funnel plot of publication bias for mir-499 rs3746444.** Log OR is plotted versus standard error of Log OR for each included study. Every circle dot represents a separate study for the indicated association (CC versus TT).
(TIF)

Table S1 **Scale for methodological quality assessment.**
(DOC)

Table S2 **Genotype frequency distributions of miRNA SNPs studied in included studies.**
(DOC)

Table S3 **Quality scores for included studies.**
(DOC)

Table S4 **Meta-analysis of studied miRNA polymorphisms after removing low scoring studies (score ≤5).**
(DOC)

Table S5 **Sensitivity analysis result for studied miRNA polymorphisms.**
(DOC)

Table S6 **Initial free energy (dG) predicted by mfold for the SNPs associated with the precursor and mature forms of human miRNAs.**
(DOC)
