# Supplementary material for: Comprehensive Review of Genetic Association Studies and Meta-Analyses on miRNA Polymorphisms and Cancer Risk
Source: PLoS One. 2012 Nov 30;7(11):e50966. doi: 10.1371/journal.pone.0050966 (PMC3511416; doi:10.1371/journal.pone.0050966)
Supplement: Table S1 — Scale for methodological quality assessment. (DOC) [file pone.0050966.s004.doc]

| **Table S1.** **Scale for methodological quality assessment.** | |
| --- | --- |
| **Criteria** | **Score** |
| ***Representativeness of cases*** |  |
| Selected from cancer registry or multiple cancer center sites | 2 |
| Selected from oncology department or cancer institute | 1 |
| Not defined | 0 |
| ***Source of controls*** |  |
| Population or community based | 2 |
| Hospital-based (cancer-free controls without any diseases) | 1.5 |
| Healthy volunteers without total description | 1 |
| Cancer-free controls with other diseases | 0.5 |
| Not defined | 0 |
| ***Ascertainment of relevant malignancy*** |  |
| Histopathologic validation | 2 |
| Patient medical record | 1 |
| Not defined | 0 |
| ***Sample size*** |  |
| >1000 | 2 |
| 200-1000 | 1 |
| <200 | 0 |
| ***Quality control of genotyping methods*** |  |
| Repetition of partial/total tested samples with a different method | 1 |
| Repetition of partial/total tested samples with the same method | 0.5 |
| Not defined | 0 |
| ***Hardy-Weinberg equilibrium (HWE)*** |  |
| Hardy-Weinberg equilibrium in control subjects | 1 |
| Hardy-Weinberg disequilibrium in control subjects for some SNPs | 0.5 |
| Hardy-Weinberg disequilibrium in control subjects | 0 |
