# Supplementary material for: Comprehensive Review of Genetic Association Studies and Meta-Analyses on miRNA Polymorphisms and Cancer Risk
Source: PLoS One. 2012 Nov 30;7(11):e50966. doi: 10.1371/journal.pone.0050966 (PMC3511416; doi:10.1371/journal.pone.0050966)
Supplement: Table S2 — Genotype frequency distributions of miRNA SNPs studied in included studies. (DOC) [file pone.0050966.s005.doc]

**Table S2. Genotype frequency distributions of miRNA SNPs studied in included studies**

| **Reference** | **Cancer** | **miRNA** | **SNP** | **rs number** | **Cases/controls** | **Genotypes (case/control)** | | | **Variant allele frequency (case/control)** |
| --- | --- | --- | --- | --- | --- | --- | --- | --- | --- |
|  |  |  |  |  |  | **Homozygote wild** | **Heterozygote** | **Homozygote variant** |  |
| Xu et al., | HCC | mir-146a | G>C | rs2910164 | 479/504 | 158/197 | 241/249 | 80/58 | 0.42/0.36 |
| Hu et al., | BC | mir-146a | G>C | rs2910164 | 1009/1093 | 329/362 | 515/551 | 165/180 | 0.42/0.42 |
|  |  | mir-149 | C>T | rs2292832 | 1009/1093 | 450/482 | 460/503 | 99/108 | 0.33/0.33 |
|  |  | mir-196a2 | C>T | rs11614913 | 1009/1093 | 239/218 | 483/517 | 287/358 | 0.48/0.44 |
|  |  | mir-499 | T>C | rs3746444 | 1009/1093 | 707/816 | 258/248 | 44/29 | 0.17/0.14 |
| Jazdzewski et al., | PTC | mir-146a | G>C | rs2910164 | 608/901 | 305/526 | 287/320 | 16/55 | 0.26/0.24 |
| Tian et al., | LC | mir-146a | G>C | rs2910164 | 1058/1035 | 360/364 | 510/502 | 188/169 | 0.42/0.41 |
|  |  | mir-149 | C>T | rs2292832 | 1058/1035 | 463/470 | 472/453 | 123/112 | 0.34/0.33 |
|  |  | mir-196a2 | C>T | rs11614913 | 1058/1035 | 253/209 | 512/519 | 293/307 | 0.48/0.45 |
|  |  | mir-499 | T>C | rs3746444 | 1058/1035 | 781/755 | 253/254 | 24/26 | 0.14/0.15 |
| Ye et al., | EC | mir-146a | G>C | rs2910164 | 328/311 | 194/178 | 118/119 | 16/14 | 0.23/0.24 |
|  |  | mir-196a2 | C>T | rs11614913 | 307/338 | 86/109 | 138/170 | 83/59 | 0.50/0.43 |
|  |  | mir-423 | A>C | rs6505162 | 329/339 | 159/111 | 129/155 | 41/73 | 0.32/0.44 |
|  |  | mir-492 | C>G | rs2289030 | 341/341 | 296/303 | 44/38 | 1/0 | 0.07/0.06 |
|  |  | mir-604 | C>T | rs2368392 | 344/314 | 179/151 | 141/141 | 24/22 | 0.27/0.29 |
|  |  | mir-608 | C>G | rs4919510 | 344/343 | 222/217 | 107/116 | 15/10 | 0.20/0.20 |
|  |  | mir-631 | CT>- | rs5745925 | 342/332 | 275/288 | 61/42 | 6/2 | 0.11/0.07 |
|  |  | mir-26a1 | C>T | rs7372209 | 343/340 | 162/176 | 147/138 | 34/26 | 0.31/0.28 |
|  |  | mir-30a | A>G | rs1358379 | 339/343 | 308/312 | 31/30 | 0/1 | 0.05/0.05 |
|  |  | mir-30c1 | C>T | rs16827546 | 342/341 | 314/322 | 28/19 | 0/0 | 0.04/0.03 |
|  |  | mir-100 | C>T | rs1834306 | 342/343 | 103/113 | 180/166 | 59/64 | 0.44/0.43 |
|  |  | mir-124a-1 | C>G | rs531564 | 342/344 | 254/267 | 75/76 | 13/1 | 0.15/0.11 |
|  |  | mir-219-1 | G>A | rs107822 | 302/316 | 180/180 | 114/119 | 8/17 | 0.22/0.24 |
|  |  |  | T>C | rs213210 | 338/344 | 284/306 | 52/38 | 2/0 | 0.08/0.06 |
|  |  | mir-373 | C>T | rs12983273 | 344/342 | 246/244 | 91/93 | 7/5 | 0.15/0.15 |
|  |  |  | C>A | rs10425222 | 330/337 | 312/320 | 18/17 | 0/0 | 0.03/0.03 |
| Catucci et al., | BC | mir-146a | G>C | rs2910164 | 1559/2147 | 860/1186 | 590/838 | 109/123 | 0.26/0.25 |
|  |  | mir-196a2 | C>T | rs11614913 | 1852/2739 | 766/1116 | 842/1246 | 244/377 | 0.36/0.37 |
|  |  | mir-499 | T>C | rs3746444 | 1579/2167 | 950/1305 | 545/742 | 84/120 | 0.23/0.23 |
| Hoffman et al., | BC | mir-196a2 | C>T | rs11614913 | 426/466 | 181/166 | 209/229 | 36/71 | 0.33/0.40 |
| Peng et al., | GC | mir-196a2 | C>T | rs11614913 | 213/213 | 76/56 | 94/107 | 43/50 | 0.42/0.49 |
| Zhou et al., | CC | mir-128 | A>G | rs11134527 | 685/713 | 268/247 | 316/339 | 101/127 | 0.38/0.42 |
| Yang et al., | BC | mir-373 | C>T | rs12983273 | 768/737 | 566/540 | 184/175 | 18/22 | 0.14/0.15 |
|  |  | mir-335 | A>G | rs3807348 | 780/736 | 257/234 | 373/368 | 150/134 | 0.43/0.43 |
|  |  | let-7a3 | G>C | rs731085 | 781/729 | 306/308 | 365/324 | 110/97 | 0.37/0.36 |
|  |  | mir-27a | A>G | rs895819 | 1189/1416 | 576/605 | 486/660 | 127/151 | 0.31/0.34 |
| Xu et al., | PC | mir-146a | G>C | rs2910164 | 251/280 | 68/54 | 135/150 | 48/76 | 0.46/0.54 |
| Qi et al., | HCC | mir-196a2 | C>T | rs11614913 | 361/391 | 82/92 | 179/197 | 100/102 | 0.48/0.49 |
| Dou et al., | Glioma | mir-196a2 | C>T | rs11614913 | 643/656 | 111/143 | 343/305 | 189/208 | 0.44/0.45 |
| Xu et al., | HCC | mir-34b/c | T>C | rs4938723 | 502/549 | 204/266 | 236/229 | 62/54 | 0.36/0.31 |
| Kim et al., | LC | mir-196a2 | C>T | rs11614913 | 654/640 | 187/155 | 305/300 | 162/185 | 0.52/0.48 |
| Christensen et al., | HNSCC | mir-196a2 | C>T | rs11614913 | 484/555 | 182/188 | 224/279 | 78/88 | 0.39/0.41 |
| Srivastava et al., | GBC | mir-196a2 | C>T | rs11614913 | 230/230 | 119/136 | 95/75 | 16/19 | 0.28/0.25 |
|  |  | mir-146a | G>C | rs2910164 | 230/224 | 129/138 | 90/81 | 11/5 | 0.24/0.20 |
|  |  | mir-499 | T>C | rs3746444 | 230/230 | 112/121 | 97/94 | 21/15 | 0.30/0.27 |
| Liu et al., | HNSCC | mir-196a2 | C>T | rs11614913 | 1109/1130 | 350/383 | 565/545 | 194/202 | 0.43/0.42 |
|  |  | mir-146a | G>C | rs2910164 | 1109/1130 | 630/655 | 411/405 | 68/70 | 0.25/0.24 |
|  |  | mir-499 | T>C | rs3746444 | 1109/1130 | 745/710 | 309/366 | 55/54 | 0.19/0.21 |
|  |  | mir-149 | G>T | rs2292832 | 1109/1130 | 580/586 | 441/445 | 88/99 | 0.28/0.28 |
| Zeng et al., | GC | mir-146a | G>C | rs2910164 | 304/304 | 62/53 | 153/132 | 89/119 | 0.39/0.46 |
| Sun et al., | GC | mir-27a | A>G | rs895819 | 304/304 | 115/145 | 135/119 | 54/40 | 0.40/0.33 |
| Guo et al., | ESCC | mir-146a | G>C | rs2910164 | 444/468 | 234/206 | 190/220 | 20/42 | 0.26/0.32 |
| Yang et al., | BC | mir-126 | G>A | rs4636297 | 2420/2741 | 957/1093 | 1136/1287 | 327/361 | 0.37/0.37 |
|  |  | mir-335 | T>A | rs41272366 | 2386/2710 | 2283/2580 | 102/130 | 1/0 | 0.02/0.02 |
| Li et al., | HCC | mir-196a2 | C>T | rs11614913 | 310/222 | 78/42 | 150/102 | 82/78 | 0.48/0.42 |
| Okubo et al., | GC | mir-196a2 | C>T | rs11614913 | 552/697 | 105/124 | 281/350 | 166/223 | 0.56/0.57 |
|  |  | mir-146a | G>C | rs2910164 | 552/697 | 73/121 | 243/322 | 236/254 | 0.65/0.60 |
|  |  | mir-499 | T>C | rs3746444 | 552/697 | 364/466 | 151/198 | 37/33 | 0.20/0.19 |
| Chen et al., | CRC | mir-196a2 | C>T | rs11614913 | 126/407 | 27/94 | 64/206 | 35/107 | 0.53/0.52 |
| Yue et al., | CC | mir-146a | G>C | rs2910164 | 447/443 | 118/87 | 224/206 | 105/150 | 0.49/0.53 |
| Mittal et al., | UBC | mir-196a2 | C>T | rs11614913 | 212/250 | 76/109 | 131/127 | 5/14 | 0.33/0.31 |
|  |  | mir-146a | G>C | rs2910164 | 212/250 | 127/135 | 79/108 | 6/7 | 0.21/0.24 |
|  |  | mir-499 | T>C | rs3746444 | 212/250 | 95/121 | 92/94 | 25/35 | 0.33/0.33 |
| Zhou et al., | CC | mir-196a2 | C>T | rs11614913 | 226/309 | 46/58 | 123/169 | 57/82 | 0.48/0.46 |
|  |  | mir-146a | G>C | rs2910164 | 226/309 | 43/34 | 113/159 | 70/116 | 0.56/0.63 |
|  |  | mir-499 | T>C | rs3746444 | 226/309 | 134/223 | 84/71 | 8/15 | 0.22/0.16 |
| Akkiz et al., | HCC | mir-196a2 | C>T | rs11614913 | 185/185 | 77/58 | 86/87 | 22/40 | 0.65/0.55 |
| Zhan et al., | CRC | mir-196a2 | C>T | rs11614913 | 252/543 | 68/113 | 128/267 | 56/163 | 0.52/0.45 |
| Hong et al., | NSCLC | mir-196a2 | C>T | rs11614913 | 406/428 | 86/96 | 224/198 | 96/134 | 0.49/0.46 |
| Permuth-Wey et al., | Glioma | mir-146a | G>C | rs2910164 | 593/614 | 345/375 | 198/214 | 50/25 | 0.25/0.21 |
| Akkiz et al., | HCC | mir-146a | G>C | rs2910164 | 222/222 | 137/144 | 75/67 | 10/11 | 0.21/0.20 |
| Zhu et al., | CRC | mir-196a2 | C>T | rs11614913 | 573/588 | 140/121 | 303/295 | 130/172 | 0.51/0.46 |
| Zhou et al., | HCC | mir-146a | G>C | rs2910164 | 186/483 | 33/71 | 86/254 | 67/158 | 0.59/0.59 |
|  |  | mir-499 | T>C | rs3746444 | 186/483 | 141/371 | 41/100 | 4/12 | 0.13/0.13 |
| Schuetz et al., | NHL  (B-cell) | mir-15a | A>G | rs9535416 | 434/454 | 122/110 | 212/239 | 100/105 | 0.47/0.49 |
|  |  | mir-1792 | A>G | rs17642969 | 522/547 | 420/445 | 95/96 | 7/6 | 0.10/0.10 |
|  |  | mir-155 | G>A | rs928883 | 522/546 | 399/430 | 108/114 | 15/2 | 0.13/0.11 |
|  | NHL  (T-cell) | mir-15a | A>G | rs2476391 | 45/547 | 38/476 | 7/66 | 0/5 | 0.08/0.07 |
|  |  | mir-1792 | A>G | rs17642969 | 45/547 | 41/445 | 4/96 | 0/6 | 0.04/0.10 |
|  |  | mir-155 | A>G | rs2829803 | 38/468 | 18/256 | 15/188 | 5/24 | 0.33/0.25 |
| Xiang et al., | HCC | mir-146a | G>C | rs2910164 | 100/100 | 27/21 | 45/46 | 28/33 | 0.51/0.56 |
|  |  | mir-499 | T>C | rs3746444 | 100/100 | 36/54 | 40/36 | 24/10 | 0.44/0.28 |
| Jedlinski et al., | BC | mir-196a2 | C>T | rs11614913 | 187/171 | 68/58 | 86/82 | 33/31 | 0.41/0.42 |
| Yang et al., | UBC | mir-146a | G>C | rs2910164 | 691/674 | 414/385 | 242/258 | 35/31 | 0.23/0.24 |
|  |  | mir-196a2 | C>T | rs11614913 | 736/731 | 255/257 | 348/342 | 133/132 | 0.42/0.41 |
|  |  | mir-423 | A>C | rs6505162 | 733/733 | 219/231 | 344/357 | 170/145 | 0.47/0.44 |
|  |  | mir-492 | C>G | rs2289030 | 739/734 | 638/655 | 98/79 | 3/0 | 0.07/0.05 |
|  |  | mir-604 | C>T | rs2368392 | 703/678 | 358/348 | 306/289 | 39/41 | 0.27/0.27 |
|  |  | mir-608 | C>G | rs4919510 | 738/737 | 457/468 | 254/241 | 27/28 | 0.21/0.20 |
|  |  | mir-631 | CT>- | rs5745925 | 720/721 | 607/609 | 110/107 | 3/5 | 0.08/0.08 |
|  |  | Let7f-2 | G>A | rs17276588 | 716/704 | 687/688 | 11/6 | 18/10 | 0.03/0.02 |
|  |  | mir-26a1 | C>T | rs7372209 | 728/728 | 362/378 | 324/288 | 42/62 | 0.28/0.28 |
|  |  | mir-30a | A>G | rs1358379 | 738/737 | 669/657 | 66/77 | 3/3 | 0.05/0.06 |
|  |  | mir-30c1 | C>T | rs16827546 | 740/736 | 694/691 | 45/45 | 1/0 | 0.03/0.03 |
|  |  | mir-100 | C>T | rs1834306 | 742/740 | 247/243 | 364/365 | 131/132 | 0.42/0.43 |
|  |  | mir-124a-1 | C>G | rs531564 | 739/740 | 562/552 | 162/181 | 15/7 | 0.13/0.13 |
|  |  | mir-219-1 | G>A | rs107822 | 705/692 | 408/389 | 259/268 | 38/35 | 0.24/0.24 |
|  |  |  | T>C | rs213210 | 737/737 | 641/644 | 96/92 | 0/1 | 0.07/0.06 |
|  |  | mir-373 | C>T | rs12983273 | 731/732 | 531/521 | 189/197 | 11/14 | 0.14/0.15 |
|  |  |  | C>A | rs10425222 | 733/728 | 676/677 | 56/49 | 1/2 | 0.04/0.04 |
| George et al., | PC | mir-196a2 | C>T | rs11614913 | 159/230 | 55/106 | 101/114 | 3/10 | 0.34/0.29 |
|  |  | mir-146a | G>C | rs2910164 | 159/230 | 76/116 | 79/107 | 4/7 | 0.27/0.26 |
|  |  | mir-499 | T>C | rs3746444 | 159/230 | 48/104 | 98/92 | 13/34 | 0.39/0.35 |
| Wang et al., | ESCC | mir-196a2 | C>T | rs11614913 | 458/489 | 148/128 | 262/250 | 48/111 | 0.39/0.48 |
| Zhang et al., | HCC | mir-128 | A>G | rs11134527 | 302/513 | 88/165 | 170/269 | 44/79 | 0.43/0.42 |
| Zhang et al., | BC | mir-605 | A>G | rs2043556 | 248/238 | 131/125 | 90/102 | 27/11 | 0.29/0.26 |
|  |  | mir-149 | C>T | rs2292832 | 245/229 | 120/92 | 102/113 | 23/24 | 0.30/0.35 |
|  |  | mir-27a | T>C | rs895819 | 244/243 | 60/75 | 144/109 | 41/59 | 0.46/0.47 |
|  |  | mir-196a2 | C>T | rs11614913 | 248/243 | 11/17 | 89/93 | 148/133 | 0.22/0.26 |
|  |  | mir-618 | C>A | rs2682818 | 244/232 | 132/130 | 99/91 | 13/11 | 0.26/0.24 |
| Pastrello et al., | BC/OC | mir-146a | G>C | rs2910164 | 101/155 | 60/90 | 36/59 | 5/6 | 0.23/0.23 |
| Vinci et al., | LC | mir-196a2 | C>T | rs11614913 | 101/129 | 35/58 | 54/61 | 12/10 | 0.39/0.31 |
|  |  | mir-146a | G>C | rs2910164 | 101/129 | 44/73 | 48/45 | 9/11 | 0.33/0.26 |
|  |  | mir-499 | T>C | rs3746444 | 101/129 | 53/70 | 41/48 | 7/11 | 0.27/0.27 |
|  |  | mir-149 | C>T | rs2292832 | 101/129 | 44/65 | 41/53 | 16/11 | 0.36/0.29 |
| Zhou et al., | GC | miR-146a | G>C | rs2910164 | 1686/1895 | 286/393 | 822/951 | 578/551 | 0.59/0.54 |
| Kim et al., | HCC | mir-196a2 | C>T | rs11614913 | 159/201 | 34/45 | 84/107 | 41/49 | 0.52/0.51 |
|  |  | mir-146a | G>C | rs2910164 | 159/201 | 14/24 | 88/103 | 57/74 | 0.71/0.62 |
|  |  | mir-499 | T>C | rs3746444 | 159/201 | 109/120 | 47/74 | 3/7 | 0.17/0.22 |
|  |  | miR-149 | C>T | rs2292832 | 159/201 | 14/21 | 64/97 | 81/83 | 0.29/0.35 |
| Smith et al., | BC | miR-423 | A>C | rs6505162 | 179/174 | 60/52 | 95/80 | 24/42 | 0.40/0.47 |
| Hishida et al., | GC | mir-146a | G>C | rs2910164 | 583/540 | 82/71 | 271/254 | 230/215 | 0.63/0.63 |
| Horikawa et al., | RCC | mir-146a | G>C | rs2910164 | 261/235 | 144/126 | 103/94 | 14/15 | 0.25/0.26 |
|  |  | mir-196a2 | C>T | rs11614913 | 276/277 | 105/101 | 126/117 | 45/59 | 0.39/0.42 |
|  |  | mir-423 | A>C | rs6505162 | 277/278 | 83/88 | 138/138 | 56/52 | 0.45/0.44 |
|  |  | mir-492 | C>G | rs2289030 | 277/277 | 248/255 | 28/20 | 1/2 | 0.05/0.04 |
|  |  | mir-604 | C>T | rs2368392 | 276/278 | 148/154 | 113/112 | 15/12 | 0.26/0.24 |
|  |  | mir-608 | C>G | rs4919510 | 275/277 | 171/178 | 91/92 | 13/7 | 0.21/0.19 |
|  |  | mir-631 | CT>- | rs5745925 | 279/278 | 225/235 | 54/40 | 0/3 | 0.10/0.08 |
|  |  | mir-26a1 | C>T | rs7372209 | 276/278 | 139/145 | 113/114 | 24/19 | 0.29/0.27 |
|  |  | mir-30a | A>G | rs1358379 | 276/278 | 252/247 | 24/28 | 0/3 | 0.04/0.06 |
|  |  | mir-30c1 | C>T | rs16827546 | 277/278 | 261/263 | 16/14 | 0/1 | 0.03/0.03 |
|  |  | mir-100 | C>T | rs1834306 | 276/277 | 91/97 | 128/133 | 57/47 | 0.44/0.41 |
|  |  | mir-124a-1 | C>G | rs531564 | 276/278 | 211/204 | 60/69 | 5/5 | 0.13/0.14 |
|  |  | mir-219-1 | G>A | rs107822 | 269/274 | 150/156 | 110/107 | 9/11 | 0.24/0.24 |
|  |  |  | T>C | rs213210 | 277/278 | 241/245 | 35/33 | 1/0 | 0.07/0.06 |
|  |  | mir-373 | C>T | rs12983273 | 276/278 | 191/207 | 81/67 | 4/4 | 0.16/0.13 |
|  |  |  | C>A | rs10425222 | 276/277 | 253/262 | 21/15 | 2/0 | 0.05/0.03 |
|  |  | let7f-2 | G>A | rs17276588 | 276/277 | 268/271 | 4/5 | 4/1 | 0.02/0.01 |
| Lung et al., | NPC | mir-146a | G>C | rs2910164 | 229/3776 | 24/497 | 88/1807 | 117/1472 | 0.70/0.63 |
| Chu et al., | OSCC | mir-146a | G>C | rs2910164 | 470/425 | 54/54 | 242/196 | 174/175 | 0.63/0.64 |
|  |  | miR-149 | C>T | rs2292832 | 470/425 | 37/26 | 88/84 | 345/315 | 0.83/0.84 |
|  |  | mir-196a2 | C>T | rs11614913 | 470/425 | 57/87 | 277/206 | 136/132 | 0.58/0.55 |
|  |  | mir-499 | T>C | rs3746444 | 470/425 | 339/356 | 119/66 | 12/3 | 0.15/0.08 |
| Bae et al., ^*^ | HCC | mir-101-1 | G>C | rs7536540 | - | - | - | - | 0.44/0.45 |
|  |  | mir101-2 | T>C | rs17803780 | - | - | - | - | 0.48/0.50 |
|  |  |  | T>C | rs12375841 | - | - | - | - | 0.30/0.27 |
|  |  | mir-338 | T>C | rs62073058 | - | - | - | - | 0.06/0.05 |

HCC: hepatocellular cancer; BC: breast cancer; GBC: gallbladder cancer; GC: gastric cancer; NSCLC: non-small cell lung carcinoma; CC: cervical cancer; LC: lung cancer; EC: esophageal cancer; PC: prostate cancer; HNSCC: head and neck squamous cell carcinoma; NHL: Non-Hodgkin lymphoma; OC: ovarian cancer; PTC: papillary thyroid carcinoma; NSCLC: non-small cell lung cancer; UBC: urinary bladder cancer; CRC: colorectal cancer; ESCC: esophageal squamous cell carcinoma; RCC: renal cell carcinoma: NPC: Nasopharyngeal Carcinoma; OSCC: oral squamous cell carcinoma.

^*^Genotype information not available even after contacting the authors.

**References:**

1. Xu T, Zhu Y, Wei Q-K, Yuan Y, Zhou F, et al. (2008) A functional polymorphism in the miR-146a gene is associated with the risk for hepatocellular carcinoma. Carcinogenesis 29: 2126-2131.

2. Hu Z, Liang J, Wang Z, Tian T, Zhou X, et al. (2009) Common genetic variants in pre-microRNAs were associated with increased risk of breast cancer in Chinese women. Hum Mutat 30: 79-84.

3. Jazdzewski K, Murray EL, Franssila K, Jarzab B, Schoenberg DR, et al. (2008) Common SNP in pre-miR-146a decreases mature miR expression and predisposes to papillary thyroid carcinoma. Proceedings of the National Academy of Sciences 105: 7269-7274.

4. Tian T, Shu Y, Chen J, Hu Z, Xu L, et al. (2009) A functional genetic variant in microRNA-196a2 is associated with increased susceptibility of lung cancer in Chinese. Cancer Epidemiol Biomarkers Prev 18: 1183-1187.

5. Ye Y, Wang KK, Gu J, Yang H, Lin J, et al. (2008) Genetic variations in microRNA-related genes are novel susceptibility loci for esophageal cancer risk. Cancer Prev Res (Phila) 1: 460-469.

6. Catucci I, Yang R, Verderio P, Pizzamiglio S, Heesen L, et al. (2010) Evaluation of SNPs in miR-146a, miR196a2 and miR-499 as low-penetrance alleles in German and Italian familial breast cancer cases. Hum Mutat 31: E1052-1057.

7. Hoffman AE, Zheng T, Yi C, Leaderer D, Weidhaas J, et al. (2009) microRNA miR-196a-2 and breast cancer: a genetic and epigenetic association study and functional analysis. Cancer Res 69: 5970-5977.

8. Peng S, Kuang Z, Sheng C, Zhang Y, Xu H, et al. (2010) Association of microRNA-196a-2 gene polymorphism with gastric cancer risk in a Chinese population. Dig Dis Sci 55: 2288-2293.

9. Zhou X, Chen X, Hu L, Han S, Qiang F, et al. (2010) Polymorphisms involved in the miR-218-LAMB3 pathway and susceptibility of cervical cancer, a case-control study in Chinese women. Gynecol Oncol 117: 287-290.

10. Yang R, Schlehe B, Hemminki K, Sutter C, Bugert P, et al. (2010) A genetic variant in the pre-miR-27a oncogene is associated with a reduced familial breast cancer risk. Breast Cancer Res Treat 121: 693-702.

11. Xu B, Feng NH, Li PC, Tao J, Wu D, et al. (2010) A functional polymorphism in Pre-miR-146a gene is associated with prostate cancer risk and mature miR-146a expression in vivo. Prostate 70: 467-472.

12. Qi P, Dou TH, Geng L, Zhou FG, Gu X, et al. (2010) Association of a variant in MIR 196A2 with susceptibility to hepatocellular carcinoma in male Chinese patients with chronic hepatitis B virus infection. Hum Immunol 71: 621-626.

13. Dou T, Wu Q, Chen X, Ribas J, Ni X, et al. (2010) A polymorphism of microRNA196a genome region was associated with decreased risk of glioma in Chinese population. J Cancer Res Clin Oncol 136: 1853-1859.

14. Xu Y, Liu L, Liu J, Zhang Y, Zhu J, et al. (2011) A potentially functional polymorphism in the promoter region of miR-34b/c is associated with an increased risk for primary hepatocellular carcinoma. Int J Cancer 128: 412-417.

15. Kim MJ, Yoo SS, Choi YY, Park JY (2010) A functional polymorphism in the pre-microRNA-196a2 and the risk of lung cancer in a Korean population. Lung Cancer 69: 127-129.

16. Christensen BC, Avissar-Whiting M, Ouellet LG, Butler RA, Nelson HH, et al. (2010) Mature microRNA sequence polymorphism in MIR196A2 is associated with risk and prognosis of head and neck cancer. Clin Cancer Res 16: 3713-3720.

17. Srivastava K, Srivastava A, Mittal B (2010) Common genetic variants in pre-microRNAs and risk of gallbladder cancer in North Indian population. J Hum Genet 55: 495-499.

18. Liu Z, Li G, Wei S, Niu J, El-Naggar AK, et al. (2010) Genetic variants in selected pre-microRNA genes and the risk of squamous cell carcinoma of the head and neck. Cancer 116: 4753-4760.

19. Zeng Y, Sun QM, Liu NN, Dong GH, Chen J, et al. (2010) Correlation between pre-miR-146a C/G polymorphism and gastric cancer risk in Chinese population. World J Gastroenterol 16: 3578-3583.

20. Sun Q, Gu H, Zeng Y, Xia Y, Wang Y, et al. (2010) Hsa-mir-27a genetic variant contributes to gastric cancer susceptibility through affecting miR-27a and target gene expression. Cancer Sci 101: 2241-2247.

21. Guo H, Wang K, Xiong G, Hu H, Wang D, et al. (2010) A functional varient in microRNA-146a is associated with risk of esophageal squamous cell carcinoma in Chinese Han. Fam Cancer 9: 599-603.

22. Yang R, Dick M, Marme F, Schneeweiss A, Langheinz A, et al. (2011) Genetic variants within miR-126 and miR-335 are not associated with breast cancer risk. Breast Cancer Res Treat 127: 549-554.

23. Li XD, Li ZG, Song XX, Liu CF (2010) A variant in microRNA-196a2 is associated with susceptibility to hepatocellular carcinoma in Chinese patients with cirrhosis. Pathology 42: 669-673.

24. Okubo M, Tahara T, Shibata T, Yamashita H, Nakamura M, et al. (2010) Association between common genetic variants in pre-microRNAs and gastric cancer risk in Japanese population. Helicobacter 15: 524-531.

25. Chen H, Sun LY, Chen LL, Zheng HQ, Zhang QF (2011) A variant in microRNA-196a2 is not associated with susceptibility to and progression of colorectal cancer in Chinese. Intern Med J.

26. Yue C, Wang M, Ding B, Wang W, Fu S, et al. (2011) Polymorphism of the pre-miR-146a is associated with risk of cervical cancer in a Chinese population. Gynecol Oncol 122: 33-37.

27. Mittal RD, Gangwar R, George GP, Mittal T, Kapoor R (2011) Investigative role of pre-microRNAs in bladder cancer patients: a case-control study in North India. DNA Cell Biol 30: 401-406.

28. Zhou B, Wang K, Wang Y, Xi M, Zhang Z, et al. (2011) Common genetic polymorphisms in pre-microRNAs and risk of cervical squamous cell carcinoma. Mol Carcinog 50: 499-505.

29. Akkiz H, Bayram S, Bekar A, Akgollu E, Ulger Y (2011) A functional polymorphism in pre-microRNA-196a-2 contributes to the susceptibility of hepatocellular carcinoma in a Turkish population: a case-control study. J Viral Hepat 18: e399-407.

30. Zhan JF, Chen LH, Chen ZX, Yuan YW, Xie GZ, et al. (2011) A functional variant in microRNA-196a2 is associated with susceptibility of colorectal cancer in a Chinese population. Arch Med Res 42: 144-148.

31. Hong YS, Kang HJ, Kwak JY, Park BL, You CH, et al. (2011) Association between microRNA196a2 rs11614913 genotypes and the risk of non-small cell lung cancer in Korean population. J Prev Med Public Health 44: 125-130.

32. Permuth-Wey J, Thompson RC, Burton Nabors L, Olson JJ, Browning JE, et al. (2011) A functional polymorphism in the pre-miR-146a gene is associated with risk and prognosis in adult glioma. J Neurooncol 105: 639-646.

33. Akkiz H, Bayram S, Bekar A, Akgollu E, Uskudar O, et al. (2011) No association of pre-microRNA-146a rs2910164 polymorphism and risk of hepatocellular carcinoma development in Turkish population: a case-control study. Gene 486: 104-109.

34. Zhu L, Chu H, Gu D, Ma L, Shi D, et al. (2011) A Functional Polymorphism in miRNA-196a2 Is Associated with Colorectal Cancer Risk in a Chinese Population. DNA Cell Biol.

35. Zhou J, Lv R, Song X, Li D, Hu X, et al. (2011) Association Between Two Genetic Variants in miRNA and Primary Liver Cancer Risk in the Chinese Population. DNA Cell Biol.

36. Schuetz JM, Daley D, Graham J, Berry BR, Gallagher RP, et al. (2012) Genetic variation in cell death genes and risk of non-hodgkin lymphoma. PLoS One 7: e31560.

37. Xiang Y, Fan S, Cao J, Huang S, Zhang LP (2012) Association of the microRNA-499 variants with susceptibility to hepatocellular carcinoma in a Chinese population. Mol Biol Rep.

38. Jedlinski DJ, Gabrovska PN, Weinstein SR, Smith RA, Griffiths LR (2011) Single nucleotide polymorphism in hsa-mir-196a-2 and breast cancer risk: a case control study. Twin Res Hum Genet 14: 417-421.

39. Yang H, Dinney CP, Ye Y, Zhu Y, Grossman HB, et al. (2008) Evaluation of genetic variants in microRNA-related genes and risk of bladder cancer. Cancer Res 68: 2530-2537.

40. George GP, Gangwar R, Mandal RK, Sankhwar SN, Mittal RD (2011) Genetic variation in microRNA genes and prostate cancer risk in North Indian population. Mol Biol Rep 38: 1609-1615.

41. Wang K, Guo H, Hu H, Xiong G, Guan X, et al. (2010) A functional variation in pre-microRNA-196a is associated with susceptibility of esophageal squamous cell carcinoma risk in Chinese Han. Biomarkers 15: 614-618.

42. Zhang LS, Liang WB, Gao LB, Li HY, Li LJ, et al. (2011) Association Between pri-miR-218 Polymorphism and Risk of Hepatocellular Carcinoma in a Han Chinese Population. DNA Cell Biol.

43. Zhang M, Jin M, Yu Y, Zhang S, Wu Y, et al. (2012) Associations of miRNA polymorphisms and female physiological characteristics with breast cancer risk in Chinese population. European Journal of Cancer Care 21: 274-280.

44. Pastrello C, Polesel J, Della Puppa L, Viel A, Maestro R (2010) Association between hsa-mir-146a genotype and tumor age-of-onset in BRCA1/BRCA2-negative familial breast and ovarian cancer patients. Carcinogenesis 31: 2124-2126.

45. Vinci S, Gelmini S, Pratesi N, Conti S, Malentacchi F, et al. (2011) Genetic variants in miR-146a, miR-149, miR-196a2, miR-499 and their influence on relative expression in lung cancers. Clin Chem Lab Med.

46. Zhou F, Zhu H, Luo D, Wang M, Dong X, et al. (2012) A Functional Polymorphism in Pre-miR-146a Is Associated with Susceptibility to Gastric Cancer in a Chinese Population. DNA Cell Biol.

47. Kim WH, Min KT, Jeon YJ, Kwon C-I, Ko KH, et al. (2012) Association study of microRNA polymorphisms with hepatocellular carcinoma in Korean population. Gene 504: 92-97.

48. Smith RA, Jedlinski DJ, Gabrovska PN, Weinstein SR, Haupt L, et al. (2012) A Genetic Variant Located in miR-423 is Associated with Reduced Breast Cancer Risk. Cancer Genomics Proteomics 9: 115-118.

49. Hishida A, Matsuo K, Goto Y, Naito M, Wakai K, et al. (2011) Combined effect of miR-146a rs2910164 G/C polymorphism and Toll-like receptor 4 +3725 G/C polymorphism on the risk of severe gastric atrophy in Japanese. Dig Dis Sci 56: 1131-1137.

50. Horikawa Y, Wood CG, Yang H, Zhao H, Ye Y, et al. (2008) Single nucleotide polymorphisms of microRNA machinery genes modify the risk of renal cell carcinoma. Clin Cancer Res 14: 7956-7962.

51. Lung RW-M, Wang X, Tong JH-M, Chau S-L, Lau K-M, et al. (2012) A single nucleotide polymorphism in microRNA-146a is associated with the risk for nasopharyngeal carcinoma. Molecular Carcinogenesis: n/a-n/a.

52. Chu Y-H, Tzeng S-L, Lin C-W, Chien M-H, Chen M-K, et al. (2012) Impacts of MicroRNA Gene Polymorphisms on the Susceptibility of Environmental Factors Leading to Carcinogenesis in Oral Cancer. PLoS ONE 7: e39777.

53. Bae JS, Kim J-H, Pasaje CFA, Cheong HS, Lee TH, et al. (2012) Association study of genetic variations in microRNAs with the risk of hepatitis B-related liver diseases. Digestive and Liver Disease.
