# Supplementary material for: Comprehensive Review of Genetic Association Studies and Meta-Analyses on miRNA Polymorphisms and Cancer Risk
Source: PLoS One. 2012 Nov 30;7(11):e50966. doi: 10.1371/journal.pone.0050966 (PMC3511416; doi:10.1371/journal.pone.0050966)
Supplement: Table S4 — Meta-analysis of studied miRNA polymorphisms after removing low scoring studies (score ≤5). (DOC) [file pone.0050966.s007.doc]

**Table S4a.** Meta-analysis of mir-146a rs2910164 polymorphism

| **Variables** | **n^a^** | **Cases/Controls** | **C-allele vs. G-allele** | | **CC vs. GG** | | **CG vs. GG** | | **Dominant**  **(CC + CG vs. GG)** | | **Recessive**  **(CC vs. CG + GG)** | |
| --- | --- | --- | --- | --- | --- | --- | --- | --- | --- | --- | --- | --- |
|  |  |  | **OR (95% CI)** | *P_Het_* | **OR**  **(95% CI)** | *P_Het_* | **OR**  **(95% CI)** | *P_Het_* | **OR**  **(95% CI)** | *P_Het_* | **OR**  **(95% CI)** | *P_Het_* |
| Total | 19 | 10122/14444 | 0.925  (0.845-1.012) | <0.001 | 0.898  (0.739-1.092) | <0.001 | 0.972  (0.915-1.032) | 0.309 | 0.959  (0.877-1.049) | 0.006 | 0.984  (0.827-1.171) | <0.001 |
| **Cancer type** | | | | | | | | | | | | |
| Hepatocellular | 2 | 701/726 | 1.222  (1.043-1.432) | 0.413 | 0.632  (0.440-0.908) | 0.317 | 1.197  (0.954-1.502) | 0.919 | 1.252  (1.008-1.554) | 0.586 | 1.426  (1.019-1.995) | 0.271 |
| Breast | 2 | 2568/3240 | 1.024  (0.945-1.109) | 0.744 | 1.095  (0.908-1.321) | 0.275 | 0.990  (0.886-1.107) | 0.632 | 1.010  (0.908-1.123) | 0.860 | 1.090  (0.916-1.298) | 0.219 |
| Other | 15 | 6853/10478 | 0.881  (0.790-0.982) | <0.001 | 0.895  (0.698-1.148) | <0.001 | 0.941  (0.873-1.015) | 0.304 | 0.913  (0.816-1.021) | 0.008 | 0.934  (0.753-1.159) | <0.001 |
| **Ethnicity** | | | | | | | | | | | | |
| Asian | 10 | 4917/8637 | 0.843  (0.730-0.973) | <0.001 | 0.743  (0.572-0.965) | <0.001 | 0.961  (0.876-1.0555) | 0.087 | 0.899  (0.758-1.066) | 0.001 | 0.874  (0.692-1.104) | <0.001 |
| Caucasian | 9 | 5205/5807 | 1.034  (0.972-1.100) | 0.349 | 1.195  (1.011-1.412) | 0.290 | 0.980  (0.905-1.061) | 0.737 | 1.007  (0.933-1.086) | 0.612 | 1.203  (1.021-1.417) | 0.296 |
| **Study design** | | | | | | | | | | | | |
| Population based | 7^*^ | 4939/9124 | 0.996  (0.859-1.154) | <0.001 | 1.205  (1.053-1.378) | 0.060 | 1.006  (0.926-1.094) | 0.980 | 1.047  (0.967-1.133) | 0.800 | 1.290  (1.041-1.599) | 0.015 |
| Hospital based | 13^*^ | 5412/9096 | 0.856  (0.759-0.965) | <0.001 | 0.758  (0.601-0.958) | 0.001 | 0.938  (0.860-1.022) | 0.117 | 0.896  (0.780-1.030) | 0.003 | 0.881  (0.700-1.109) | <0.001 |

Random effects model was used when *P* value of Q for heterogeneity test (*P_Het_*) <0.05; otherwise, fixed effect model was used.

^a^ Number of studies involved

^*^The study by Lung et al., has both hospital based and population based controls.

OR: odds ratio; CI: confidence interval

**Table S4b.** Meta-analysis of mir-196a2 rs11614913 polymorphism

| **Variables** | **n^a^** | **Cases/Controls** | **T-allele vs. C-allele** | | **TT vs. CC** | | **TC vs. CC** | | **Dominant**  **(TT + TC vs. CC)** | | **Recessive**  **(TT vs. TC + CC)** | |
| --- | --- | --- | --- | --- | --- | --- | --- | --- | --- | --- | --- | --- |
|  |  |  | **OR (95% CI)** | *P_Het_* | **OR**  **(95% CI)** | *P_Het_* | **OR**  **(95% CI)** | *P_Het_* | **OR**  **(95% CI)** | *P_Het_* | **OR**  **(95% CI)** | *P_Het_* |
| Total | 25 | 12545/14011 | 0.926  (0.871-0.984) | <0.001 | 0.828  (0.722-0.949) | <0.001 | 0.999  (0.913-1.093) | 0.001 | 0.950  (0.866-1.043) | <0.001 | 0.840  (0.758-0.931) | <0.001 |
| **Cancer type** | | | | | | | | | | | | |
| Breast | 4 | 3262/3969 | 0.912  (0.786-1.059) | 0.009 | 0.799  (0.569-1.122) | 0.006 | 0.941  (0.850-1.042) | 0.375 | 0.911  (0.828-1.003) | 0.079 | 0.858  (0.672-1.096) | 0.013 |
| Lung | 3 | 2118/2103 | 0.874  (0.803-0.952) | 0.854 | 0.770  (0.649-0.912) | 0.895 | 0.896  (0.769-1.044) | 0.098 | 0.848  (0.735-0.979) | 0.289 | 0.830  (0.725-0.949) | 0.281 |
| Colorectal | 3 | 951/1538 | 0.848  (0.754-0.954) | 0.223 | 0.690  (0.543-0.876) | 0.150 | 0.886  (0.719-1.091) | 0.636 | 0.813  (0.667-0.990) | 0.351 | 0.751  (0.621-0.909) | 0.209 |
| Hepatocellular | 3 | 856/798 | 0.811  (0.608-1.082) | 0.016 | 0.658  (0.368-1.177) | 0.017 | 0.870  (0.685-1.105) | 0.505 | 0.813  (0.649-1.018) | 0.144 | 0.736  (0.469-1.155) | 0.026 |
| Other | 12 | 5358/5603 | 0.988  (0.897-1.088) | 0.001 | 0.925  (0.733-1.167) | <0.001 | 1.111  (0.953-1.295) | 0.001 | 1.067  (0.920-1.239) | 0.001 | 0.876  (0.727-1.055) | <0.001 |
| **Ethnicity** | | | | | | | | | | | | |
| Asian | 15 | 7007/7682 | 0.897  (0.832-0.968) | 0.002 | 0.806  (0.675-0.962) | <0.001 | 0.985  (0.854-1.136) | 0.001 | 0.919  (0.799-1.058) | <0.001 | 0.816  (0.726-0.916) | 0.004 |
| Caucasian | 10 | 5538/6329 | 0.970  (0.879-1.069) | 0.002 | 0.866  (0.698-1.076) | 0.001 | 1.015  (0.940-1.096) | 0.112 | 0.995  (0.885-1.119) | 0.028 | 0.874  (0.717-1.067) | 0.001 |
| **Study design** | | | | | | | | | | | | |
| Population based | 10^*^ | 6174/6983 | 0.892  (0.818-0.973) | 0.005 | 0.753  (0.619-0.914) | 0.002 | 0.946  (0.875-1.022) | 0.148 | 0.902  (0.838-0.971) | 0.072 | 0.786  (0.660-0.936) | <0.001 |
| Hospital based | 16^*^ | 6797/7494 | 0.934  (0.857-1.018) | <0.001 | 0.846  (0.697-1.027) | <0.001 | 1.011  (0.887-1.153) | 0.001 | 0.963  (0.839-1.106) | <0.001 | 0.852  (0.746-0.974) | 0.001 |

Random effects model was used when *P* value of Q-test for heterogeneity test (*P_Het_*) <0.05; otherwise, fixed effect model was used.

^a^ Number of studies involved

^*^The study by Hoffman et al., has both hospital based and population based controls.

OR: odds ratio; CI: confidence interval

**Table S4c.** Meta-analysis of mir-499 rs3746444 polymorphism

| **Variables** | **n^a^** | **Cases/Controls** | **C-allele vs. T-allele** | | **CC vs. TT** | | **CT vs. TT** | | **Dominant**  **(CC + CT vs. TT)** | | **Recessive**  **(CC vs. CT + TT)** | |
| --- | --- | --- | --- | --- | --- | --- | --- | --- | --- | --- | --- | --- |
|  |  |  | **OR (95% CI)** | *P_Het_* | **OR**  **(95% CI)** | *P_Het_* | **OR**  **(95% CI)** | *P_Het_* | **OR**  **(95% CI)** | *P_Het_* | **OR**  **(95% CI)** | *P_Het_* |
| Total | 8 | 5893/6639 | 1.142  (0.982-1.329) | <0.001 | 1.086  (0.912-1.293) | 0.139 | 1.175  (0.973-1.419) | <0.001 | 1.178  (0.980-1.415) | <0.001 | 1.066  (0.898-1.266) | 0.170 |
| **Cancer type** | | | | | | | | | | | | |
| Breast | 2 | 2588/3260 | 1.115  (0.878-1.417) | 0.017 | 1.257  (0.701-2.255) | 0.036 | 1.067  (0.952-1.196) | 0.163 | 1.079  (0.967-1.203) | 0.056 | 1.111  (0.869-1.421) | 0.050 |
| Other | 6 | 3305/3379 | 1.166  (0.933-1.457) | <0.001 | 1.044  (0.817-1.334) | 0.268 | 1.233  (0.917-1.658) | <0.001 | 1.223  (0.922-1.621) | <0.001 | 1.025  (0.806-1.304) | 0.279 |
| **Ethnicity** | | | | | | | | | | | | |
| Asian | 4 | 2763/2862 | 1.332  (1.011-1.756) | <0.001 | 1.349  (0.973-1.869) | 0.068 | 1.397  (1.012-1.928) | <0.001 | 1.400  (1.020-1.922) | <0.001 | 1.275  (0.922-1.764) | 0.071 |
| Caucasian | 4 | 3130/3777 | 0.974  (0.899-1.055) | 0.275 | 0.995  (0.809-1.223) | 0.684 | 0.960  (0.867-1.064) | 0.096 | 0.963  (0.874-1.062) | 0.147 | 0.994  (0.812-1.218) | 0.639 |
| **Study design** | | | | | | | | | | | | |
| Population based | 4 | 3876/4525 | 1.054  (0.975-1.139) | 0.054 | 1.121  (0.903-1.393) | 0.127 | 1.045  (0.949-1.151) | 0.420 | 1.055  (0.963-1.157) | 0.164 | 1.105  (0.892-1.369) | 0.177 |
| Hospital based | 4 | 2017/2114 | 1.246  (0.866-1.794) | <0.001 | 1.022  (0.761-1.373) | 0.168 | 1.372  (0.836-2.250) | <0.001 | 1.340  (0.840-2.137) | <0.001 | 0.999  (0.748-1.334) | 0.163 |

Random effects model was used when *P* value of Q-test for heterogeneity test (*P_Het_*) <0.05; otherwise, fixed effect model was used.

^a^ Number of studies involved

OR: odds ratio; CI: confidence interval

**Table S4d.** Meta-analysis of mir-149 rs2292832 polymorphism

| **Variables** | **n^a^** | **Cases/Controls** | **T-allele vs. C-allele** | | **TT vs. CC** | | **TC vs. CC** | | **Dominant**  **(TT + TC vs. CC)** | | **Recessive**  **(TT vs. TC + CC)** | |
| --- | --- | --- | --- | --- | --- | --- | --- | --- | --- | --- | --- | --- |
|  |  |  | **OR (95% CI)** | *P_Het_* | **OR**  **(95% CI)** | *P_Het_* | **OR**  **(95% CI)** | *P_Het_* | **OR**  **(95% CI)** | *P_Het_* | **OR**  **(95% CI)** | *P_Het_* |
| Total | 5 | 3891/3912 | 0.982  (0.917-1.053) | 0.429 | 0.998  (0.848-1.174) | 0.566 | 0.978  (0.886-1.079) | 0.303 | 0.976  (0.889-1.072) | 0.271 | 0.981  (0.853-1.129) | 0.911 |
| **Cancer type** | | | | | | | | | | | | |
| Breast | 2 | 1254/1322 | 0.949  (0.845-1.067) | 0.167 | 0.931  (0.709-1.222) | 0.418 | 0.919  (0.781-1.082) | 0.106 | 0.921  (0.789-1.077) | 0.101 | 0.971  (0.750-1.259) | 0.737 |
| Other | 3 | 2637/2590 | 1.000  (0.918-1.090) | 0.492 | 1.037  (0.847-1.270) | 0.386 | 1.013  (0.895-1.146) | 0.501 | 1.008  (0.897-1.133) | 0.439 | 0.986  (0.834-1.165) | 0.648 |
| **Ethnicity** | | | | | | | | | | | | |
| Asian | 4 | 2782/2782 | 0.987  (0.910-1.071) | 0.286 | 1.039  (0.858-1.257) | 0.505 | 0.967  (0.859-1.089) | 0.191 | 0.973  (0.869-1.089) | 0.161 | 1.006  (0.859-1.179) | 0.907 |
| Caucasian | 1 | 1109/1130 | 0.969  (0.851-1.104) | 1.000 | 0.898  (0.659-1.224) | 1.000 | 1.001  (0.841-1.192) | 1.000 | 0.982  (0.832-1.160) | 1.000 | 0.898  (0.665-1.212) | 1.000 |
| **Study design** | | | | | | | | | | | | |
| Population based | 3 | 2312/2357 | 0.996  (0.914-1.086) | 0.185 | 1.014  (0.832-1.235) | 0.482 | 0.979  (0.867-1.105) | 0.144 | 0.985  (0.877-1.106) | 0.119 | 1.024  (0.849-1.235) | 0.802 |
| Hospital based | 2 | 1579/1555 | 0.957  (0.853-1.074) | 0.688 | 0.965  (0.726-1.284) | 0.234 | 0.976  (0.826-1.154) | 0.323 | 0.960  (0.819-1.124) | 0.363 | 0.930  (0.753-1.149) | 0.742 |

Random effects model was used when *P* value of Q-test for heterogeneity test (*P_Het_*) <0.05; otherwise, fixed effect model was used.

^a^ Number of studies involved

OR: odds ratio; CI: confidence interval

**References:**

1. Lung RW-M, Wang X, Tong JH-M, Chau S-L, Lau K-M, et al. (2012) A single nucleotide polymorphism in microRNA-146a is associated with the risk for nasopharyngeal carcinoma. Molecular Carcinogenesis: n/a-n/a.

2. Hoffman AE, Zheng T, Yi C, Leaderer D, Weidhaas J, et al. (2009) microRNA miR-196a-2 and breast cancer: a genetic and epigenetic association study and functional analysis. Cancer Res 69: 5970-5977.
