# Supplementary material for: Comprehensive Review of Genetic Association Studies and Meta-Analyses on miRNA Polymorphisms and Cancer Risk
Source: PLoS One. 2012 Nov 30;7(11):e50966. doi: 10.1371/journal.pone.0050966 (PMC3511416; doi:10.1371/journal.pone.0050966)
Supplement: Table S5 — Sensitivity analysis result for studied miRNA polymorphisms. (DOC) [file pone.0050966.s008.doc]

**Table S5a:** Sensitivity analysis result for mir-146a rs2910164 (CC vs. GG)

| **Study** | **OR** | **95% CI** | | ***p* value** |
| --- | --- | --- | --- | --- |
|  |  | **Lower limit** | **Upper limit** |  |
| Jazdzewski | 0.940 | 0.795 | 1.112 | 0.470 |
| Tian | 0.908 | 0.759 | 1.086 | 0.288 |
| Xu | 0.942 | 0.797 | 1.114 | 0.487 |
| Xu | 0.940 | 0.794 | 1.113 | 0.472 |
| Hu | 0.914 | 0.763 | 1.096 | 0.332 |
| Ye | 0.915 | 0.770 | 1.087 | 0.311 |
| Catucci | 0.903 | 0.757 | 1.077 | 0.258 |
| Srivastava | 0.903 | 0.764 | 1.068 | 0.235 |
| Liu | 0.914 | 0.766 | 1.091 | 0.318 |
| Zeng | 0.934 | 0.787 | 1.109 | 0.434 |
| Guo | 0.947 | 0.803 | 1.116 | 0.515 |
| Okubo | 0.893 | 0.755 | 1.056 | 0.186 |
| Yue | 0.947 | 0.804 | 1.116 | 0.516 |
| Mittal | 0.919 | 0.775 | 1.089 | 0.329 |
| Zhou | 0.939 | 0.794 | 1.112 | 0.467 |
| Permuth-Wey | 0.885 | 0.753 | 1.041 | 0.141 |
| Akkiz | 0.918 | 0.773 | 1.089 | 0.325 |
| Zhou | 0.919 | 0.772 | 1.094 | 0.340 |
| Xiang | 0.927 | 0.781 | 1.100 | 0.386 |
| Yang | 0.913 | 0.767 | 1.087 | 0.307 |
| George | 0.919 | 0.775 | 1.090 | 0.331 |
| Pastrello | 0.914 | 0.771 | 1.084 | 0.302 |
| Vinci | 0.911 | 0.768 | 1.080 | 0.283 |
| Chu | 0.915 | 0.768 | 1.090 | 0.320 |
| Horikawa | 0.922 | 0.776 | 1.094 | 0.351 |
| Kim | 0.909 | 0.766 | 1.078 | 0.273 |
| Lung | 0.894 | 0.755 | 1.058 | 0.192 |

**Table S5b:** Sensitivity analysis result for mir-196a2 rs11614913 (TT vs. CC)

| **Study** | **OR** | **95% CI** | | ***p* value** |
| --- | --- | --- | --- | --- |
|  |  | **Lower limit** | **Upper limit** |  |
| Hu | 0.852 | 0.748 | 0.971 | 0.017 |
| Tian | 0.849 | 0.745 | 0.969 | 0.015 |
| Ye | 0.824 | 0.731 | 0.929 | 0.002 |
| Catucci | 0.841 | 0.736 | 0.961 | 0.011 |
| Hoffman | 0.864 | 0.764 | 0.977 | 0.019 |
| Peng | 0.853 | 0.752 | 0.969 | 0.014 |
| Qi | 0.838 | 0.737 | 0.952 | 0.007 |
| Dou | 0.834 | 0.734 | 0.947 | 0.005 |
| Kim | 0.852 | 0.749 | 0.970 | 0.015 |
| Christensen | 0.843 | 0.741 | 0.960 | 0.010 |
| Srivastava | 0.844 | 0.743 | 0.958 | 0.009 |
| Liu | 0.837 | 0.735 | 0.953 | 0.007 |
| Li | 0.857 | 0.756 | 0.972 | 0.017 |
| Okubo | 0.845 | 0.742 | 0.962 | 0.011 |
| Chen | 0.839 | 0.739 | 0.953 | 0.007 |
| Mittal | 0.851 | 0.751 | 0.965 | 0.012 |
| Zhou | 0.845 | 0.744 | 0.961 | 0.010 |
| Akkiz | 0.861 | 0.761 | 0.974 | 0.017 |
| Zhan | 0.858 | 0.757 | 0.974 | 0.017 |
| Hong | 0.848 | 0.745 | 0.965 | 0.012 |
| Zhu | 0.856 | 0.753 | 0.972 | 0.017 |
| Jedlinski | 0.845 | 0.744 | 0.959 | 0.009 |
| Yang | 0.839 | 0.737 | 0.955 | 0.008 |
| George | 0.849 | 0.749 | 0.962 | 0.010 |
| Wang | 0.873 | 0.778 | 0.980 | 0.021 |
| Zhang | 0.836 | 0.738 | 0.946 | 0.005 |
| Vinci | 0.836 | 0.739 | 0.947 | 0.005 |
| Chu | 0.827 | 0.732 | 0.934 | 0.002 |
| Horikawa | 0.850 | 0.748 | 0.966 | 0.013 |
| Kim | 0.840 | 0.740 | 0.954 | 0.007 |

**Table S5c:** Sensitivity analysis result for mir-499 rs3746444 (CC vs. TT)

| **Study** | **OR** | **95% CI** | | ***p* value** |
| --- | --- | --- | --- | --- |
|  |  | **Lower limit** | **Upper limit** |  |
| Hu | 1.068 | 0.908 | 1.256 | 0.425 |
| Tian | 1.145 | 0.976 | 1.342 | 0.097 |
| Catucci | 1.193 | 0.996 | 1.429 | 0.055 |
| Srivastava | 1.107 | 0.946 | 1.296 | 0.203 |
| Liu | 1.154 | 0.977 | 1.364 | 0.092 |
| Okubo | 1.094 | 0.931 | 1.286 | 0.276 |
| Mittal | 1.142 | 0.974 | 1.339 | 0.103 |
| Zhou | 1.129 | 0.967 | 1.318 | 0.125 |
| Zhou | 1.132 | 0.969 | 1.323 | 0.119 |
| Xiang | 1.080 | 0.924 | 1.263 | 0.331 |
| George | 1.140 | 0.974 | 1.334 | 0.102 |
| Vinci | 1.131 | 0.969 | 1.321 | 0.119 |
| Chu | 1.102 | 0.944 | 1.286 | 0.218 |
| Kim | 1.136 | 0.973 | 1.326 | 0.106 |

**Table S5d:** Sensitivity analysis result for mir-149 rs2292832 (TT vs. CC)

| **Study** | **OR** | **95% CI** | | ***p* value** |
| --- | --- | --- | --- | --- |
|  |  | **Lower limit** | **Upper limit** |  |
| Hu | 1.007 | 0.843 | 1.201 | 0.942 |
| Tian | 0.958 | 0.800 | 1.147 | 0.642 |
| Liu | 1.035 | 0.869 | 1.234 | 0.697 |
| Zhang | 1.019 | 0.871 | 1.193 | 0.810 |
| Vinci | 0.976 | 0.835 | 1.139 | 0.754 |
| Chu | 0.984 | 0.842 | 1.149 | 0.835 |
| Kim | 1.025 | 0.874 | 1.202 | 0.764 |

**References**

1. Jazdzewski K, Murray EL, Franssila K, Jarzab B, Schoenberg DR, et al. (2008) Common SNP in pre-miR-146a decreases mature miR expression and predisposes to papillary thyroid carcinoma. Proceedings of the National Academy of Sciences 105: 7269-7274.

2. Tian T, Shu Y, Chen J, Hu Z, Xu L, et al. (2009) A functional genetic variant in microRNA-196a2 is associated with increased susceptibility of lung cancer in Chinese. Cancer Epidemiol Biomarkers Prev 18: 1183-1187.

3. Xu B, Feng NH, Li PC, Tao J, Wu D, et al. (2010) A functional polymorphism in Pre-miR-146a gene is associated with prostate cancer risk and mature miR-146a expression in vivo. Prostate 70: 467-472.

4. Xu T, Zhu Y, Wei Q-K, Yuan Y, Zhou F, et al. (2008) A functional polymorphism in the miR-146a gene is associated with the risk for hepatocellular carcinoma. Carcinogenesis 29: 2126-2131.

5. Hu Z, Liang J, Wang Z, Tian T, Zhou X, et al. (2009) Common genetic variants in pre-microRNAs were associated with increased risk of breast cancer in Chinese women. Hum Mutat 30: 79-84.

6. Ye Y, Wang KK, Gu J, Yang H, Lin J, et al. (2008) Genetic variations in microRNA-related genes are novel susceptibility loci for esophageal cancer risk. Cancer Prev Res (Phila) 1: 460-469.

7. Catucci I, Yang R, Verderio P, Pizzamiglio S, Heesen L, et al. (2010) Evaluation of SNPs in miR-146a, miR196a2 and miR-499 as low-penetrance alleles in German and Italian familial breast cancer cases. Hum Mutat 31: E1052-1057.

8. Srivastava K, Srivastava A, Mittal B (2010) Common genetic variants in pre-microRNAs and risk of gallbladder cancer in North Indian population. J Hum Genet 55: 495-499.

9. Liu Z, Li G, Wei S, Niu J, El-Naggar AK, et al. (2010) Genetic variants in selected pre-microRNA genes and the risk of squamous cell carcinoma of the head and neck. Cancer 116: 4753-4760.

10. Zeng Y, Sun QM, Liu NN, Dong GH, Chen J, et al. (2010) Correlation between pre-miR-146a C/G polymorphism and gastric cancer risk in Chinese population. World J Gastroenterol 16: 3578-3583.

11. Guo H, Wang K, Xiong G, Hu H, Wang D, et al. (2010) A functional varient in microRNA-146a is associated with risk of esophageal squamous cell carcinoma in Chinese Han. Fam Cancer 9: 599-603.

12. Okubo M, Tahara T, Shibata T, Yamashita H, Nakamura M, et al. (2010) Association between common genetic variants in pre-microRNAs and gastric cancer risk in Japanese population. Helicobacter 15: 524-531.

13. Yue C, Wang M, Ding B, Wang W, Fu S, et al. (2011) Polymorphism of the pre-miR-146a is associated with risk of cervical cancer in a Chinese population. Gynecol Oncol 122: 33-37.

14. Mittal RD, Gangwar R, George GP, Mittal T, Kapoor R (2011) Investigative role of pre-microRNAs in bladder cancer patients: a case-control study in North India. DNA Cell Biol 30: 401-406.

15. Zhou B, Wang K, Wang Y, Xi M, Zhang Z, et al. (2011) Common genetic polymorphisms in pre-microRNAs and risk of cervical squamous cell carcinoma. Mol Carcinog 50: 499-505.

16. Permuth-Wey J, Thompson RC, Burton Nabors L, Olson JJ, Browning JE, et al. (2011) A functional polymorphism in the pre-miR-146a gene is associated with risk and prognosis in adult glioma. J Neurooncol 105: 639-646.

17. Akkiz H, Bayram S, Bekar A, Akgollu E, Uskudar O, et al. (2011) No association of pre-microRNA-146a rs2910164 polymorphism and risk of hepatocellular carcinoma development in Turkish population: a case-control study. Gene 486: 104-109.

18. Zhou J, Lv R, Song X, Li D, Hu X, et al. (2011) Association Between Two Genetic Variants in miRNA and Primary Liver Cancer Risk in the Chinese Population. DNA Cell Biol.

19. Xiang Y, Fan S, Cao J, Huang S, Zhang LP (2012) Association of the microRNA-499 variants with susceptibility to hepatocellular carcinoma in a Chinese population. Mol Biol Rep.

20. Yang H, Dinney CP, Ye Y, Zhu Y, Grossman HB, et al. (2008) Evaluation of genetic variants in microRNA-related genes and risk of bladder cancer. Cancer Res 68: 2530-2537.

21. George GP, Gangwar R, Mandal RK, Sankhwar SN, Mittal RD (2011) Genetic variation in microRNA genes and prostate cancer risk in North Indian population. Mol Biol Rep 38: 1609-1615.

22. Pastrello C, Polesel J, Della Puppa L, Viel A, Maestro R (2010) Association between hsa-mir-146a genotype and tumor age-of-onset in BRCA1/BRCA2-negative familial breast and ovarian cancer patients. Carcinogenesis 31: 2124-2126.

23. Vinci S, Gelmini S, Pratesi N, Conti S, Malentacchi F, et al. (2011) Genetic variants in miR-146a, miR-149, miR-196a2, miR-499 and their influence on relative expression in lung cancers. Clin Chem Lab Med.

24. Chu Y-H, Tzeng S-L, Lin C-W, Chien M-H, Chen M-K, et al. (2012) Impacts of MicroRNA Gene Polymorphisms on the Susceptibility of Environmental Factors Leading to Carcinogenesis in Oral Cancer. PLoS ONE 7: e39777.

25. Horikawa Y, Wood CG, Yang H, Zhao H, Ye Y, et al. (2008) Single nucleotide polymorphisms of microRNA machinery genes modify the risk of renal cell carcinoma. Clin Cancer Res 14: 7956-7962.

26. Kim WH, Min KT, Jeon YJ, Kwon C-I, Ko KH, et al. (2012) Association study of microRNA polymorphisms with hepatocellular carcinoma in Korean population. Gene 504: 92-97.

27. Lung RW-M, Wang X, Tong JH-M, Chau S-L, Lau K-M, et al. (2012) A single nucleotide polymorphism in microRNA-146a is associated with the risk for nasopharyngeal carcinoma. Molecular Carcinogenesis: n/a-n/a.

28. Hoffman AE, Zheng T, Yi C, Leaderer D, Weidhaas J, et al. (2009) microRNA miR-196a-2 and breast cancer: a genetic and epigenetic association study and functional analysis. Cancer Res 69: 5970-5977.

29. Peng S, Kuang Z, Sheng C, Zhang Y, Xu H, et al. (2010) Association of microRNA-196a-2 gene polymorphism with gastric cancer risk in a Chinese population. Dig Dis Sci 55: 2288-2293.

30. Qi P, Dou TH, Geng L, Zhou FG, Gu X, et al. (2010) Association of a variant in MIR 196A2 with susceptibility to hepatocellular carcinoma in male Chinese patients with chronic hepatitis B virus infection. Hum Immunol 71: 621-626.

31. Dou T, Wu Q, Chen X, Ribas J, Ni X, et al. (2010) A polymorphism of microRNA196a genome region was associated with decreased risk of glioma in Chinese population. J Cancer Res Clin Oncol 136: 1853-1859.

32. Kim MJ, Yoo SS, Choi YY, Park JY (2010) A functional polymorphism in the pre-microRNA-196a2 and the risk of lung cancer in a Korean population. Lung Cancer 69: 127-129.

33. Christensen BC, Avissar-Whiting M, Ouellet LG, Butler RA, Nelson HH, et al. (2010) Mature microRNA sequence polymorphism in MIR196A2 is associated with risk and prognosis of head and neck cancer. Clin Cancer Res 16: 3713-3720.

34. Li XD, Li ZG, Song XX, Liu CF (2010) A variant in microRNA-196a2 is associated with susceptibility to hepatocellular carcinoma in Chinese patients with cirrhosis. Pathology 42: 669-673.

35. Chen H, Sun LY, Chen LL, Zheng HQ, Zhang QF (2011) A variant in microRNA-196a2 is not associated with susceptibility to and progression of colorectal cancer in Chinese. Intern Med J.

36. Zhan JF, Chen LH, Chen ZX, Yuan YW, Xie GZ, et al. (2011) A functional variant in microRNA-196a2 is associated with susceptibility of colorectal cancer in a Chinese population. Arch Med Res 42: 144-148.

37. Hong YS, Kang HJ, Kwak JY, Park BL, You CH, et al. (2011) Association between microRNA196a2 rs11614913 genotypes and the risk of non-small cell lung cancer in Korean population. J Prev Med Public Health 44: 125-130.

38. Zhu L, Chu H, Gu D, Ma L, Shi D, et al. (2011) A Functional Polymorphism in miRNA-196a2 Is Associated with Colorectal Cancer Risk in a Chinese Population. DNA Cell Biol.

39. Jedlinski DJ, Gabrovska PN, Weinstein SR, Smith RA, Griffiths LR (2011) Single nucleotide polymorphism in hsa-mir-196a-2 and breast cancer risk: a case control study. Twin Res Hum Genet 14: 417-421.

40. Wang K, Guo H, Hu H, Xiong G, Guan X, et al. (2010) A functional variation in pre-microRNA-196a is associated with susceptibility of esophageal squamous cell carcinoma risk in Chinese Han. Biomarkers 15: 614-618.

41. Zhang M, Jin M, Yu Y, Zhang S, Wu Y, et al. (2012) Associations of miRNA polymorphisms and female physiological characteristics with breast cancer risk in Chinese population. European Journal of Cancer Care 21: 274-280.
