# Supplementary material for: Comprehensive Review of Genetic Association Studies and Meta-Analyses on miRNA Polymorphisms and Cancer Risk
Source: PLoS One. 2012 Nov 30;7(11):e50966. doi: 10.1371/journal.pone.0050966 (PMC3511416; doi:10.1371/journal.pone.0050966)
Supplement: Table S6 — Initial free energy (dG) predicted by mfold for the SNPs associated with the precursor and mature forms of human miRNAs. (DOC) [file pone.0050966.s009.doc]

**Table S6. Initial free energy (dG) predicted by mfold for the SNPs associated with the precursor and mature forms of human miRNAs**

| 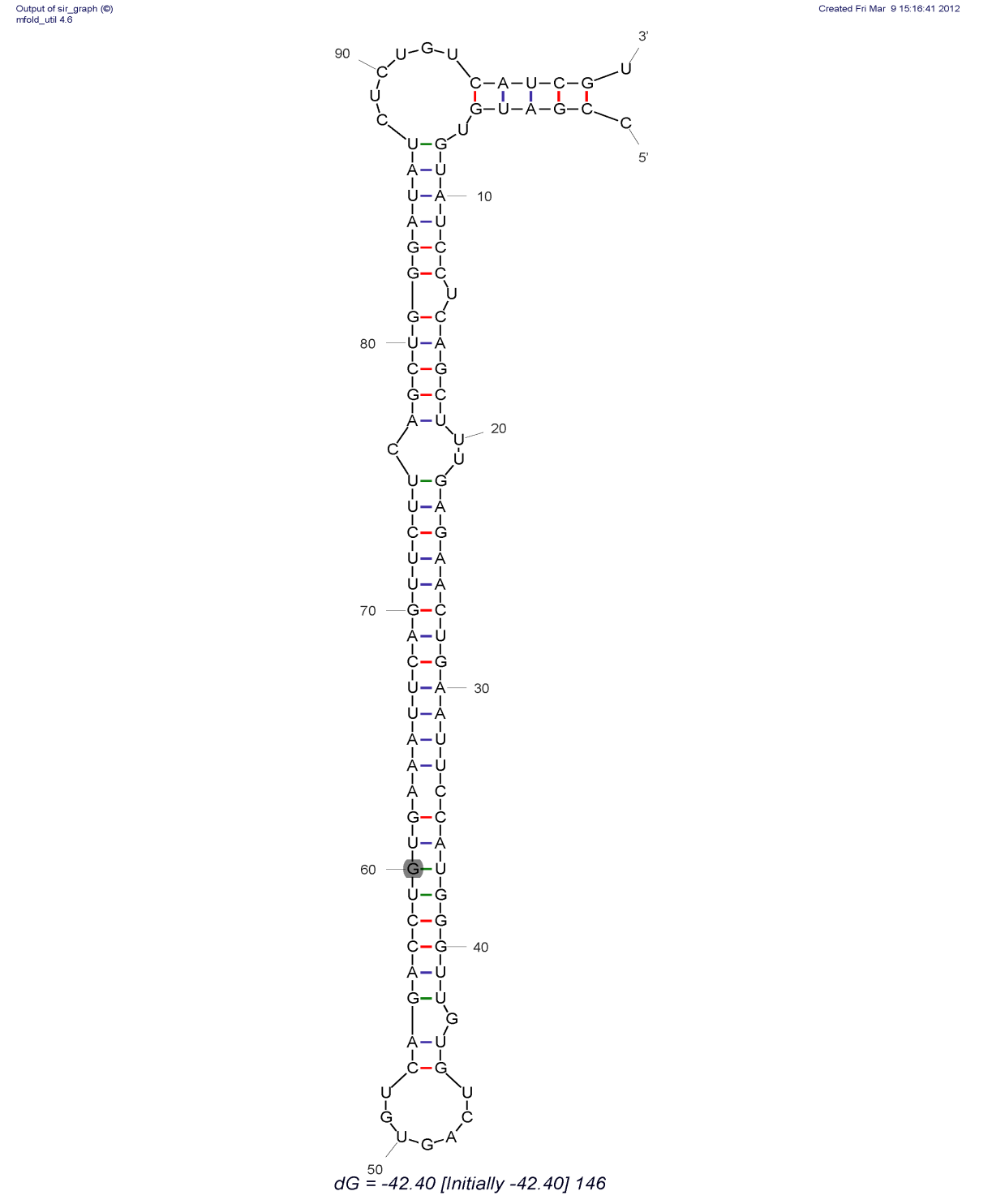 | 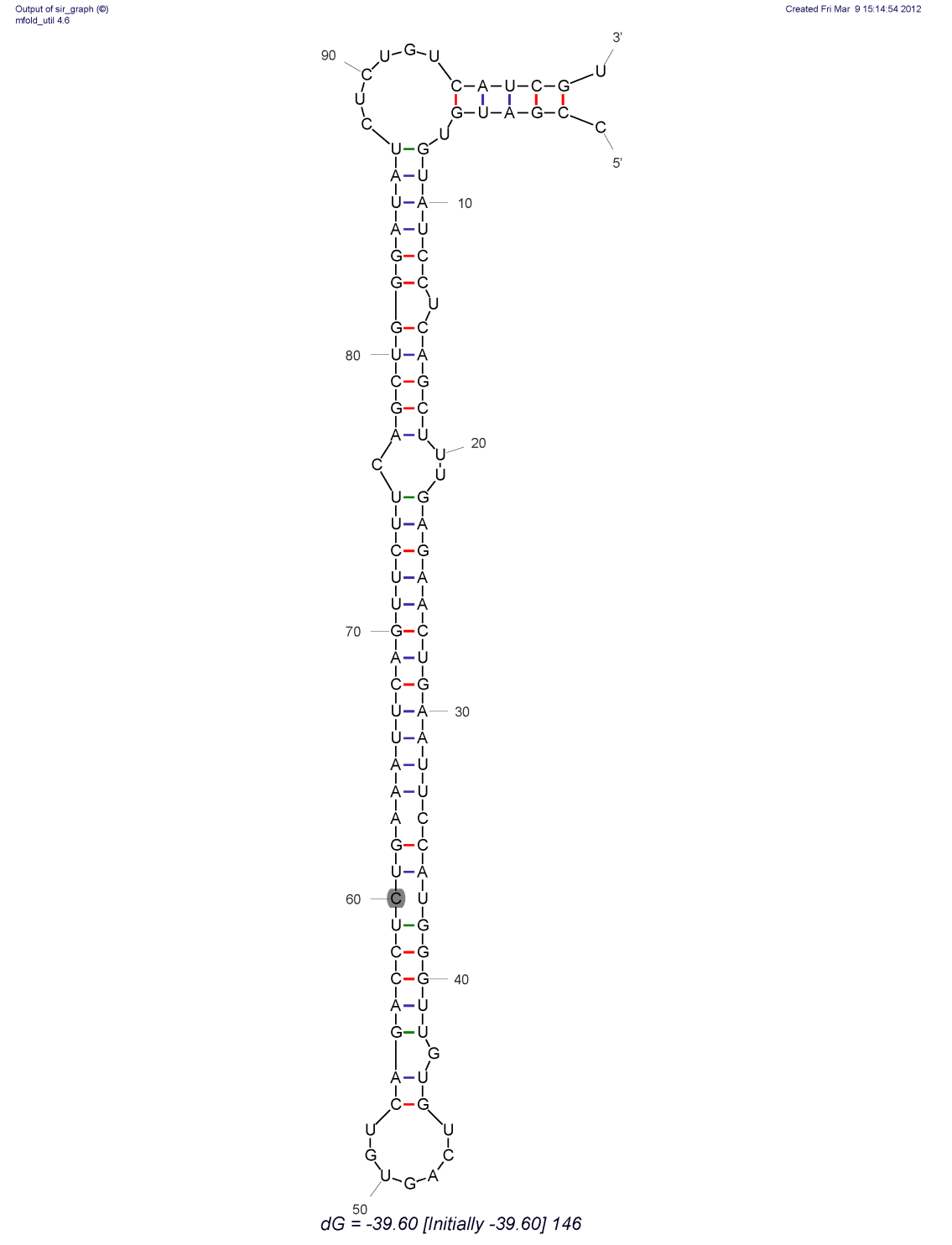 | 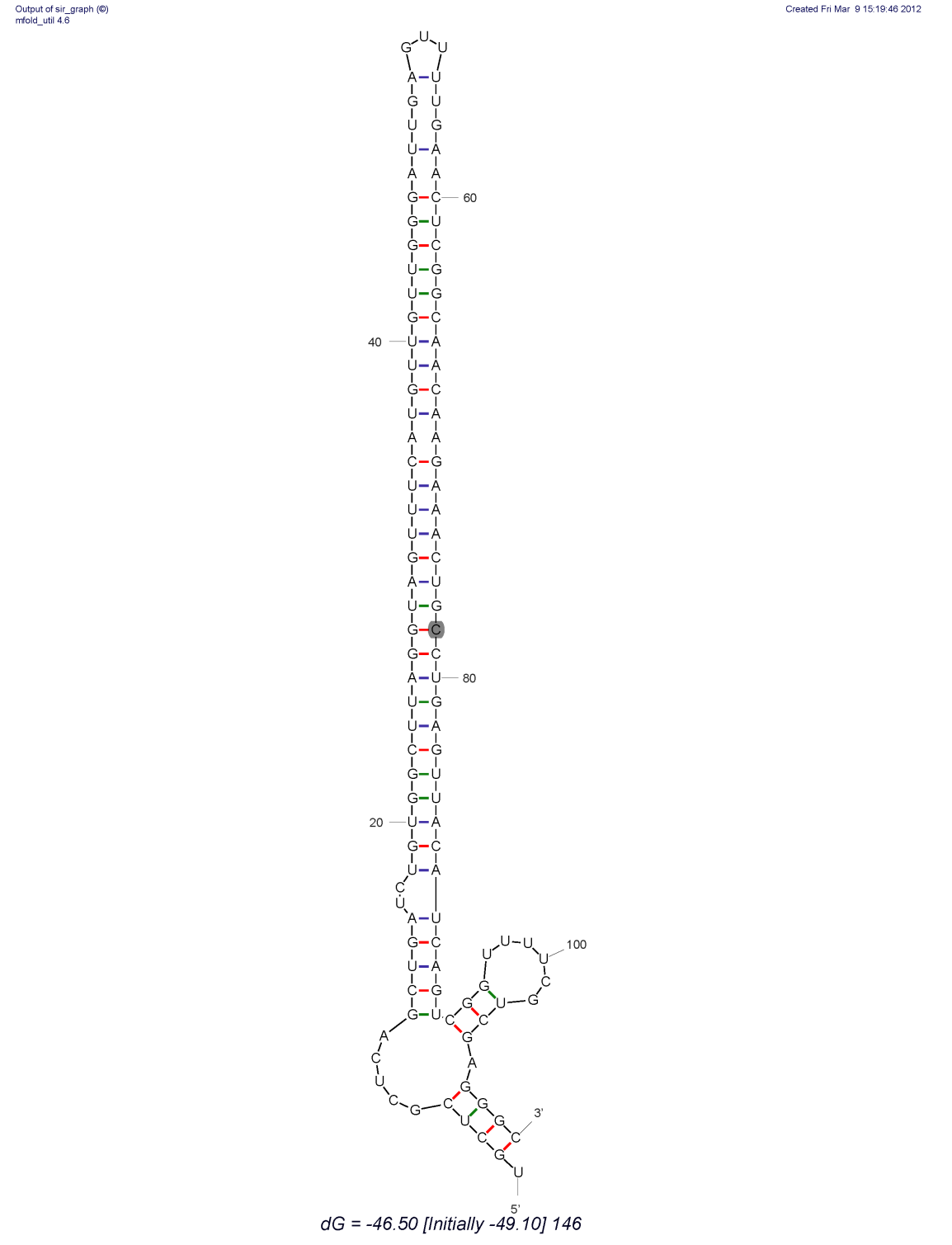 | 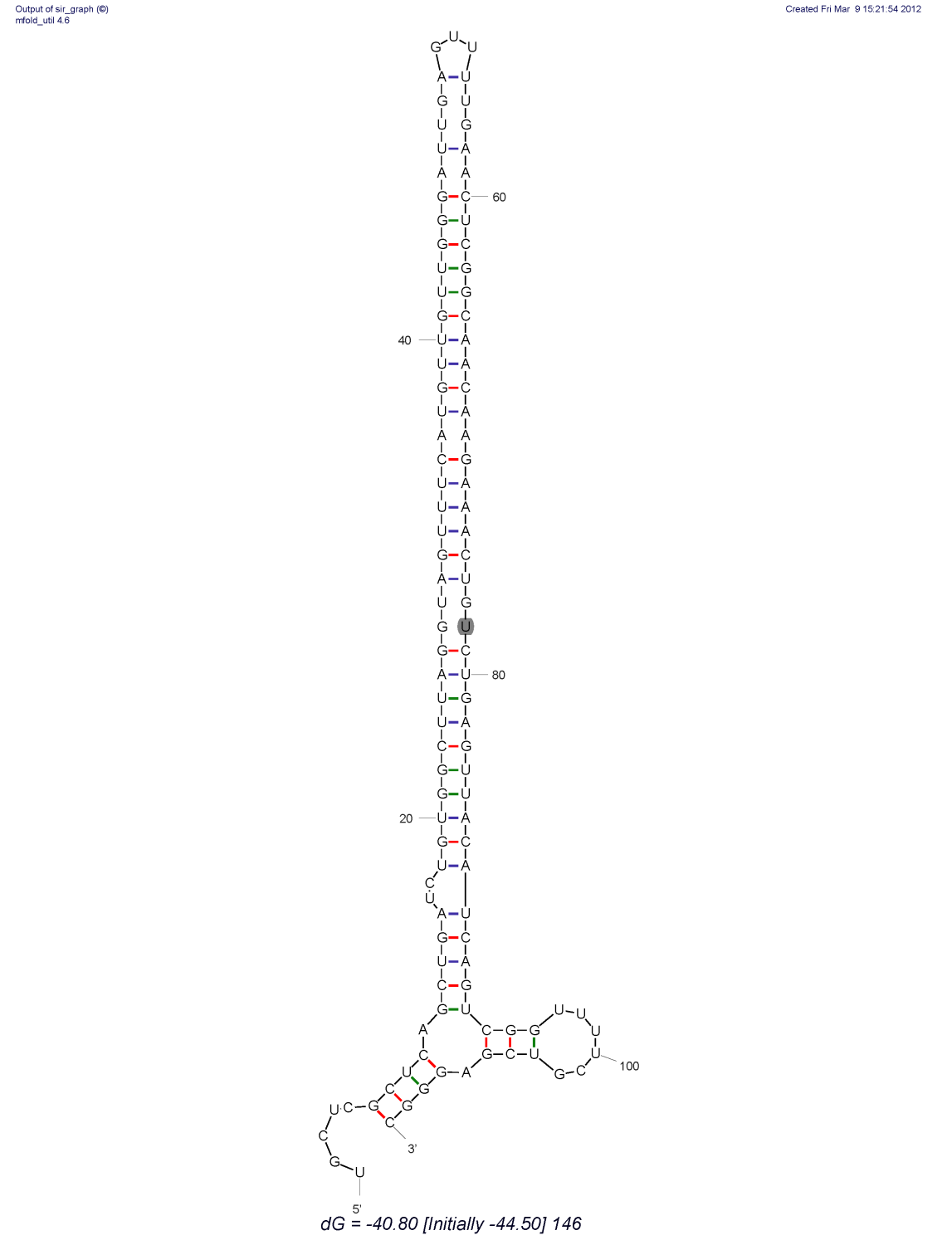 | 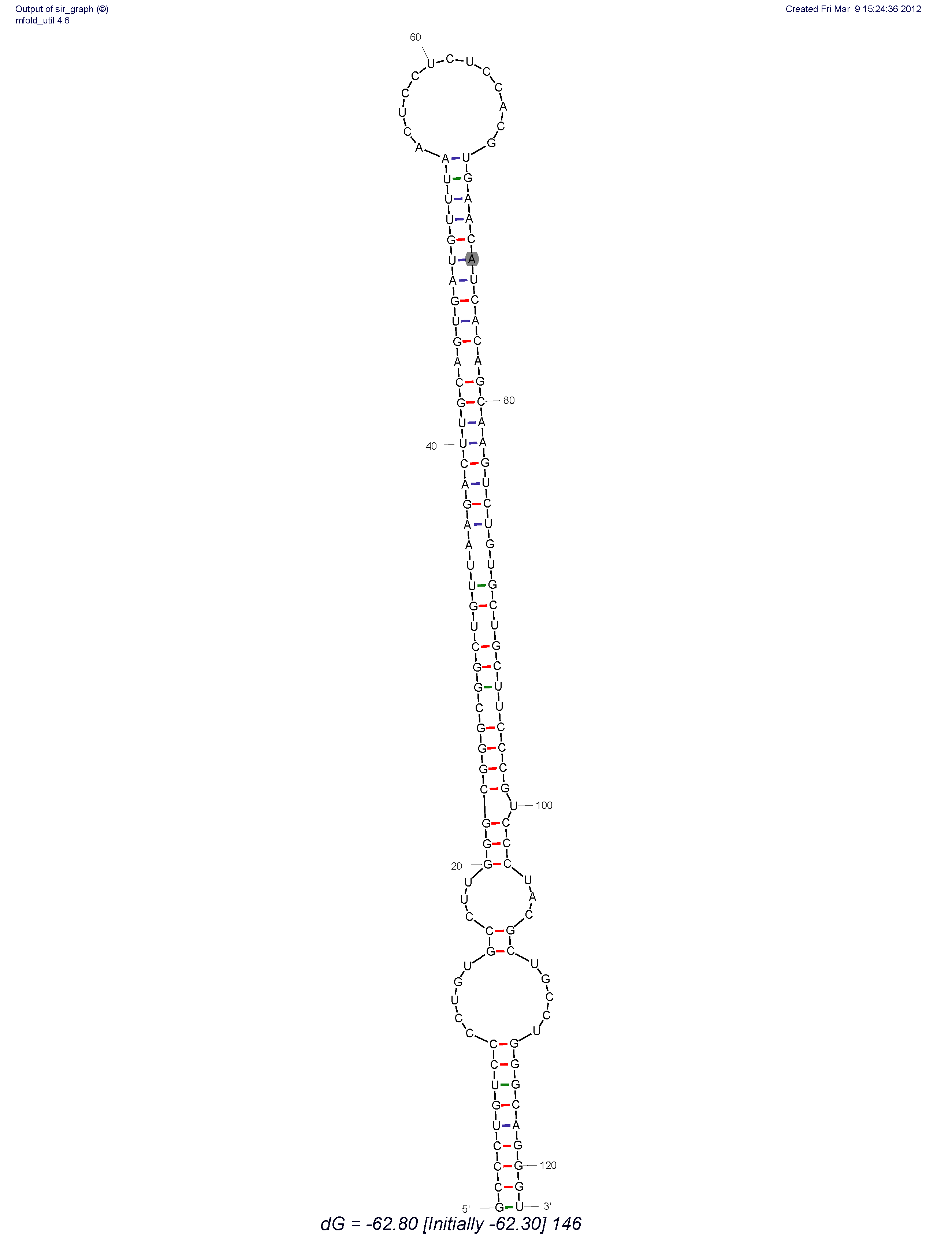 | 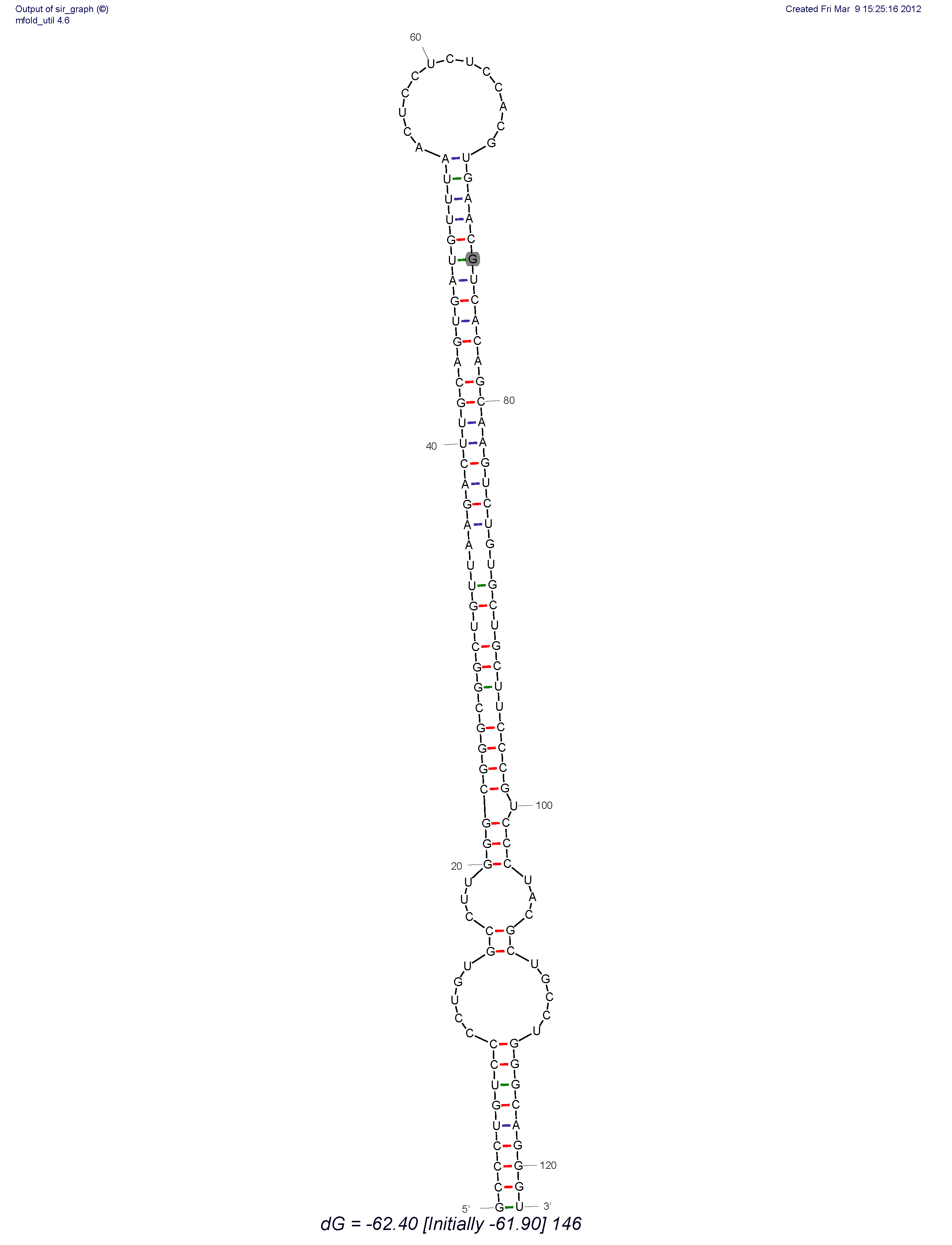 | 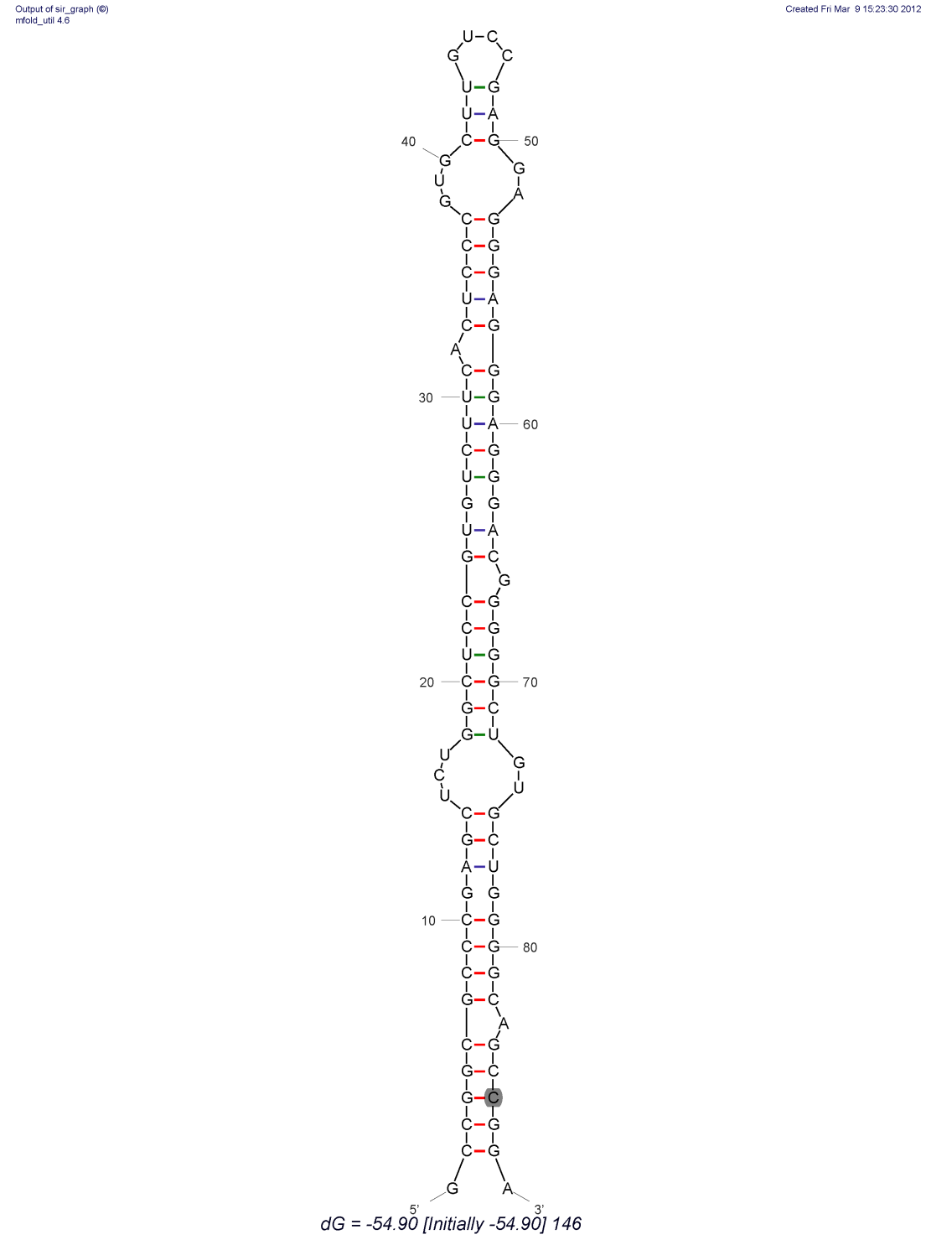 | 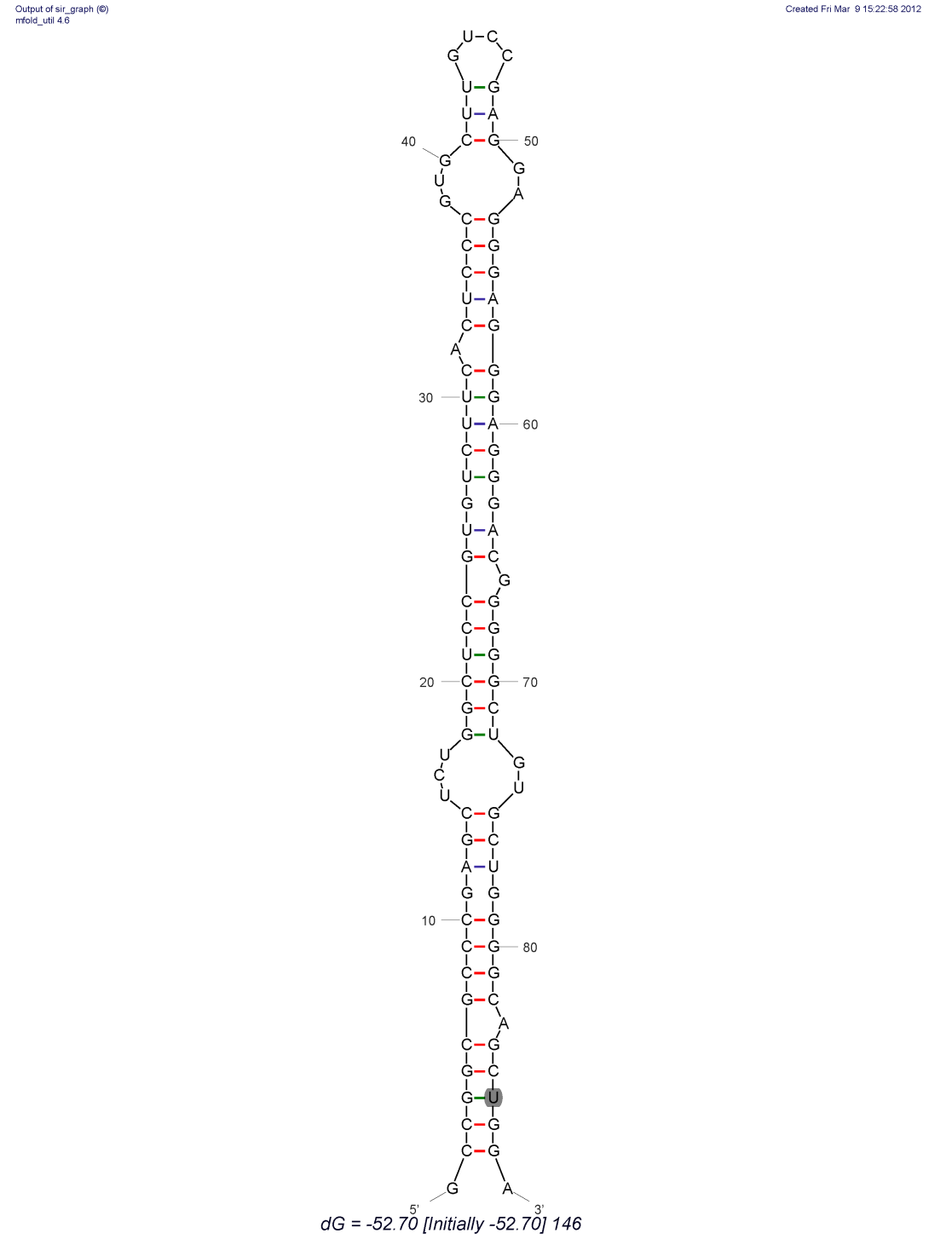 |
| --- | --- | --- | --- | --- | --- | --- | --- |
| hsa-mir-146a rs2910164 : G | hsa-mir-146a rs2910164 : C | hsa-mir-196a2 rs11614913: C | hsa-mir-196a2 rs11614913: T | hsa-mir-499 rs3746444 : T | hsa-mir-499 rs3746444 : C | hsa-mir-149 rs2292832 : C | hsa-mir-149 rs2292832 : T |
| CCGAUGUGUAUCCUCAGCUUUGAGAACUGAAUUCCAUGGGUUGUGUCAGUGUCAGACCU**C**UGAAAUUCAGUUCUUCAGCUGGGAUAUCUCUGUCAUCGU | | UGCUCGCUCAGCUGAUCUGUGGCUUAGGUAGUUUCAUGUUGUUGGGAUUGAGUUUUGAACUCGGCAACAAGAAACUG**C**CUGAGUUACAUCAGUCGGUUUUCGUCGAGGGC | | GCCCUGUCCCCUGUGCCUUGGGCGGGCGGCUGUUAAGACUUGCAGUGAUGUUUAACUCCUCUCCACGUGAAC**A**UCACAGCAAGUCUGUGCUGCUUCCCGUCCCUACGCUGCCUGGGCAGGGU | | GCCGGCGCCCGAGCUCUGGCUCCGUGUCUUCACUCCCGUGCUUGUCCGAGGAGGGAGGGAGGGACGGGGGCUGUGCUGGGGCAGC**U**GGA | |
| Predicted initial dG = -42.40 kcal/mol | Predicted initial dG = -39.60 kcal/mol | Predicted initial  dG = -49.10 kcal/mol | Predicted initial  dG = -44.50 kcal/mol | Predicted initial  dG = -62.30 kcal/mol | Predicted initial  dG = -61.90 kcal/mol | Predicted initial dG = -54.90 kcal/mol | Predicted initial dG = -52.70 kcal/mol |

Bold bases denote the site of polymorphism
